# PEGylated Positively Charged Fluorescent Silica Nanoparticles for Enhanced Gene Delivery in Canola (*Brassica napus*)

**DOI:** 10.1021/acsomega.6c03943

**Published:** 2026-09-03

**Authors:** Zhila Osmani, Muhammad Amirul Islam, Lipu Wang, Rui Wen, Wei Xiao, Guanqun Chen, Marianna Kulka

**Affiliations:** † Department of Medical Microbiology and Immunology, 12357 University of Alberta, Edmonton, Alberta T6G 2E1, Canada; ‡ 6356 Quantum and Nanotechnologies Research Centre, National Research Council Canada, Edmonton, Alberta T6G 2M9, Canada; § Department of Biochemistry, Microbiology & Immunology, 7235 University of Saskatchewan, Saskatoon, Saskatchewan S7N 5E5, Canada; ∥ Department of Plant Sciences, University of Saskatchewan, Saskatoon, Saskatchewan S7N 5A8, Canada; ⊥ Saskatoon Research and Development Centre, 98679 Agriculture and Agri-Food Canada, Saskatoon, Saskatchewan S7N 0X2, Canada; # Department of Agricultural, Food & Nutritional Science, Faculty of Agricultural, Life and Environmental Sciences, 3158 University of Alberta, Edmonton, Alberta T6G 2E1, Canada; ∇ Applied Virology Institute, Li Ka Shing Centre for Health Innovation, University of Alberta, Edmonton, Alberta T6G 2C5, Canada

## Abstract

Genetic transformation of *Brassica napus* (canola) using conventional methods remains limited by low efficiency and tissue-specific barriers, but nanoparticle (NP)-mediated delivery offers a promising complementary approach to overcome these limitations. In this study, a PEGylated, positively charged fluorescent silica nanoparticle (SiO_2_NP–TMAC/FITC/PEG) was developed and evaluated for DNA delivery and real-time tracking in canola protoplasts and microspores. This multifunctional platform integrates quaternary ammonium groups for efficient DNA binding and enhanced cellular interaction, FITC for fluorescence-based monitoring, and PEG for improved stability, reduced cytotoxicity, and enhanced delivery performance. Together, these features enable a robust and versatile nanocarrier system capable of efficiently delivering plasmid DNA while allowing simultaneous tracking of cellular internalization and transformation efficacy. Protoplasts were isolated from both leaf and hypocotyl tissues, and microspores were obtained from unopened floral buds. Fluorescent reporter plasmids were complexed with functional SiO_2_NPs, and uptake efficiency and cell viability were assessed using fluorescein diacetate and trypan blue staining. Among the four nanocarrier designs, PEG surface modification improved DNA uptake and cell viability compared with non-PEGylated SiO_2_ nanoparticles. The multifunctional SiO_2_NP–TMAC/FITC/PEG platform showed the best delivery and tracking performance, demonstrating a strong synergistic effect and highlighting its promise for efficient gene delivery. Among protoplast sources, hypocotyl-derived protoplasts exhibited superior transformation efficiency and more sustained expression than leaf-derived protoplasts, while in microspores, developmental stages strongly influenced uptake, and larger buds (3–4 mm) were more amenable to NP transformation. Transient transformation results demonstrate the feasibility of NP-mediated gene delivery in canola and provide practical insights for optimizing dose, exposure time, and cell type. These data will allow for the integration of NP delivery with established transformation methods and could further enhance efficiency and facilitate future efforts toward stable integration in *B. napus*.

## Graphical abstract

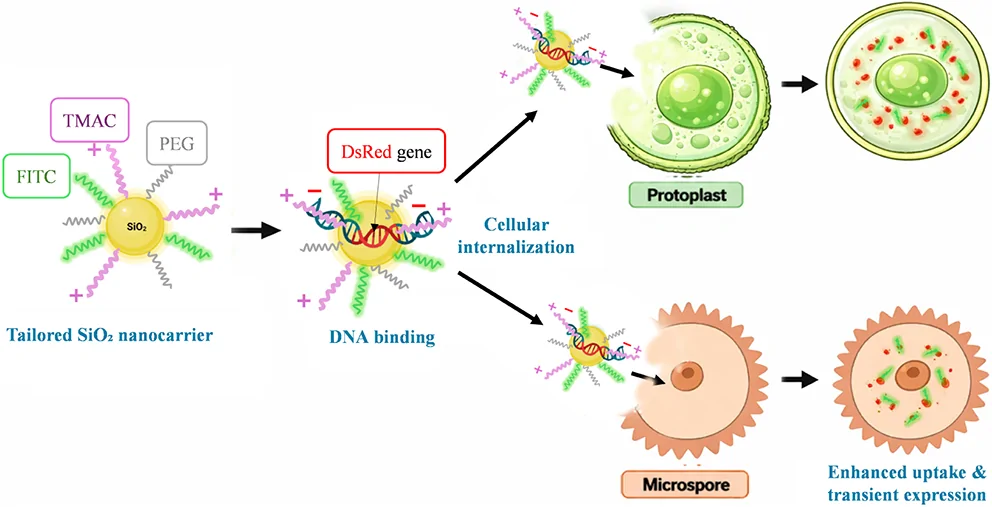

## 1 Introduction

Canola (*Brassica napus*), a major oilseed crop worldwide,^1^ is a key target for genetic improvement efforts aimed at enhancing disease resistance, stress tolerance, quality, oil content, and yield traits.^2–5^ Agrobacterium-mediated transformation of hypocotyl tissues, biolistic delivery to microspores, and PEG-mediated transformation of protoplasts represent the most commonly used approaches for gene delivery in canola.^6,7^ Despite their widespread use, these methods face several bottlenecks that limit the efficient plant genetic transformation. In particular, Agrobacterium-mediated delivery in canola remains challenging due to strong genotype dependence, tissue-specific responses, and generally low and variable transformation efficiencies.^8,9^

Microspores are immature male gametophytes that possess high cellular plasticity and can be induced to develop into haploid or doubled haploid embryos under appropriate culture conditions.^10^ This allows for the rapid fixation of genetic traits and the efficient development of homozygous lines, which are highly beneficial in plant breeding and genetic studies.^11,12^ A major biological barrier to microspore transformation is the presence of a highly resistant exine and intine, both of which prevent passive molecular diffusion across the cell surface.^10,13^ The biolistic method, widely applied for the transformation of microspores and immature embryos, also has major limitations. These include extensive physical damage to target cells caused by particle bombardment, random integration events leading to unstable expression, and frequent rearrangements or truncations of introduced DNA. In addition, biolistic transformation is often accompanied by low reproducibility and genotype dependence, which restricts its broader application in crop biotechnology.^14–16^

Protoplasts, on the other hand, are plant cells that have had their cell walls enzymatically removed, making them permeable to foreign DNA and other macromolecules.^17^ Therefore, protoplasts are a flexible model for studying early cellular responses to transformation and are often used in transient expression assays.^17,18^ Polyethylene glycol (PEG)-mediated delivery has been widely used for protoplast transformation;^19–21^ however, the overall efficiency of PEG-mediated DNA delivery remains relatively low. This limited efficiency is due to several factors, including osmotic stress, reduced cell viability at high concentrations, and enzymatic degradation of DNA during extended cytoplasmic exposure before nuclear entry.^22–24^ Positively charged cationic polymers are well-known to facilitate gene delivery by forming electrostatic complexes with negatively charged nucleic acids, resulting in condensation into nanosized particles that interact efficiently with the negatively charged components of the cell surface and are internalized via endocytosis.^25^ Their ability to condense DNA helps shield the DNA from nuclease degradation, and these polymers can be engineered with a wide range of sizes and architectures, allowing high loading capacity, tunable structures, and versatile structural modifications to support targeted delivery.^25–27^ Once internalized, the cationic charges facilitate endosomal membrane destabilization, allowing the DNA–polymer complex to escape into the cytosol. However, excessive cationic charge can increase cytotoxicity, induce nonspecific interactions, and lead to strong, irreversible DNA–polymer binding. Such irreversible binding hinders cytosolic release and often directs the complex to lysosomes, where the DNA is enzymatically degraded. The efficiency of polymer-mediated transformation is also strongly influenced by multiple experimental parameters, including polymer/DNA ratio, concentration, and incubation time, requiring optimization for each cell type.^28^

In recent years, nanomaterials have emerged as promising tools in plant biotechnology, offering novel approaches for the delivery of biomolecules into plant cells with high precision and minimal cytotoxicity. In addition, nanomaterials can protect biomolecules from enzymatic degradation.^29–32^ Silica nanoparticles (SiO_2_NPs) are broadly classified as solid (nonporous) or porous, with solid particles offering high structural integrity, uniformity, and excellent physicochemical stability. Their biocompatibility and tunable surface chemistry make them ideal candidates for gene delivery.^33,34^ Despite promising results with mesoporous silica nanoparticles and carbon nanotubes in other species, the use of solid SiO_2_NPs for DNA delivery in *Brassica napus* protoplasts and microspores has not been systematically explored.^31,35^

Although native SiO_2_NPs possess a negatively charged surface that limits direct interaction with DNA, surface functionalization strategies have significantly enhanced their gene delivery capabilities.^33^ This study integrates the functionalities of cationic groups, PEG, and FITC on a single solid SiO_2_NP nanocarrier platform, yielding SiO_2_NP–TMAC/PEG/FITC for efficient plasmid DNA (pDNA) delivery and tracking in *B. napus* cells. Similar to cationic polymers, quaternary ammonium-functionalized SiO_2_NPs are presumed to bind DNA through electrostatic interactions, associate with the cell surface, and are likely internalized via endocytosis. A properly balanced cationic surface charge is thought to facilitate DNA release into the cytosol, whereas an excessive positive charge may increase cytotoxicity, promote nonspecific interactions, and enhance lysosomal trapping. PEG is one of the most commonly used surface modifiers because it confers colloidal stability, reduces cytotoxicity, and acts as a stealth layer to minimize nonspecific cellular interactions. PEG also facilitates DNA transformation by destabilizing the cell membrane, a process known as PEG-mediated transformation.^36,37^ In addition, it provides a steric barrier that protects NP-bound DNA from enzymatic degradation and masks excessive positive charge to reduce toxicity.^38^ By preventing strong, irreversible DNA-NP complexation, PEG potentially can promote DNA release and support nuclear internalization once the complex enters the cell and escapes from endosomes.^36,38^

Meanwhile, FITC modification of SiO_2_NPs enables tracking of DNA–NP uptake and monitoring the fate of both the carriers and DNA inside cells. By optimizing parameters such as NP concentration, PEGylation efficiency, and DNA-NP complex formation, we aim to establish an effective and biocompatible NP-based gene delivery system suitable for plant transformation applications. Gene delivery was successfully demonstrated in both canola microspores and protoplasts, indicating that this platform can be applied to plant cells with intact cell walls as well as to wall-free cell systems.

## 2 Results

### 2.1 Synthesis and Characterization of Surface-Tailored SiO_2_NPs

SiO_2_NPs were functionalized with two functional groups, −NH_2_ and −N­(CH_3_)_3_^+^, to impart DNA-binding capacity and enable further covalent modification; the resulting material is denoted as SiO_2_NP-APTMS/TMAC. Incorporation of quaternary ammonium groups shifted the ζ-potential from negative (−43.8 mV ± 12.5) to strongly positive (+45.8 mV ± 12.4), confirming successful surface functionalization.

Dynamic light scattering revealed an increase in particle size from 13.0 ± 6.8 nm to 30.3 ± 15.2 nm (Figure 1a), reflecting functional group attachment and hydration. SEM images showed that functionalization reduced aggregation, leading to more uniform particle distribution. FTIR, NMR, and XPS confirmed the presence of both −NH_2_ and −N­(CH_3_)_3_^+^ groups, while TGA indicated ∼14% organic content associated with surface functional groups (please see SEM, TGA, XPS, and NMR experimental details and results in our previous publication).^39^ Further modification with FITC dye was achieved by forming thiourea bonds between the isothiocyanate group of FITC and the primary amine on SiO_2_NP-APTMS/TMAC, yielding SiO_2_NP-TMAC/FITC conjugates, as indicated by the yellow color and green fluorescence of the purified NPs. Conjugation was confirmed by ATR-FTIR (Figure 2b), which showed suppression of the primary amine stretching bands at 3380 and 3266 cm^–1^ and the bending band at 1641 cm^–1^, consistent with their consumption during thiourea bond formation with FITC. The appearance of aromatic C–H stretching at 3018 cm^–1^ and the phenolic O–H band at 3656 cm^–1^ further confirms the presence of FITC conjugated to the SiO_2_NP–APTMS/TMAC surface. The hydrodynamic diameter increased to 42.5 ± 60.7 nm (Figure 1b), while the ζ-potential (45.5 ± 4.9 mV) remained nearly identical to that of SiO_2_NP-APTMS/TMAC. HOOC-PEG-OCH_3_ was conjugated to SiO_2_NP-APTMS/TMAC through EDC-mediated coupling of its carboxyl group with surface amines, producing SiO_2_NP-TMAC/PEG conjugates. ATR-FTIR analysis provided evidence of successful PEG conjugation, as the characteristic primary amine stretching signals at 3380 and 3266 cm^–1^, along with the bending vibration at 1641 cm^–1^, were markedly diminished, indicative of their participation in the amide linkage formed with PEG. Enhanced C–H stretching (2800–2990 cm^–1^) and bending (1340 cm^–1^) were also observed, along with a shift of the Si–O–Si peak from 1040 cm^–1^ to 1072 cm^–1^, attributed to C–O–C vibrations of PEG ether groups (Figure 2c). The hydrodynamic diameter increased to 50.0 ± 42.8 nm (Figure 1c) compared to SiO_2_NP-APTMS/TMAC, reflecting the larger particle size from PEG chain grafting. In contrast, the ζ-potential decreased to 21.6 ± 4.2 mV. Conjugation of FITC and PEG to SiO_2_NP-APTMS/TMAC via thiourea and amide bond formation yielded the PEGylated positively charged fluorescent silica nanoparticle (SiO_2_NP-TMAC/PEG/FITC). ATR-FTIR spectra displayed changes similar to those seen after PEG coupling alone, without distinct FITC peaks (Figure 2d); however, the yellow coloration and green fluorescence of the purified particles confirmed FITC incorporation alongside PEG. The hydrodynamic diameter increased to 63.2 ± 27.8 nm, with some aggregates reaching 368.5 ± 149.7 nm (Figure 1d), while the ζ-potential decreased to 27.3 ± 3.5 mV.

**Figure 1:**
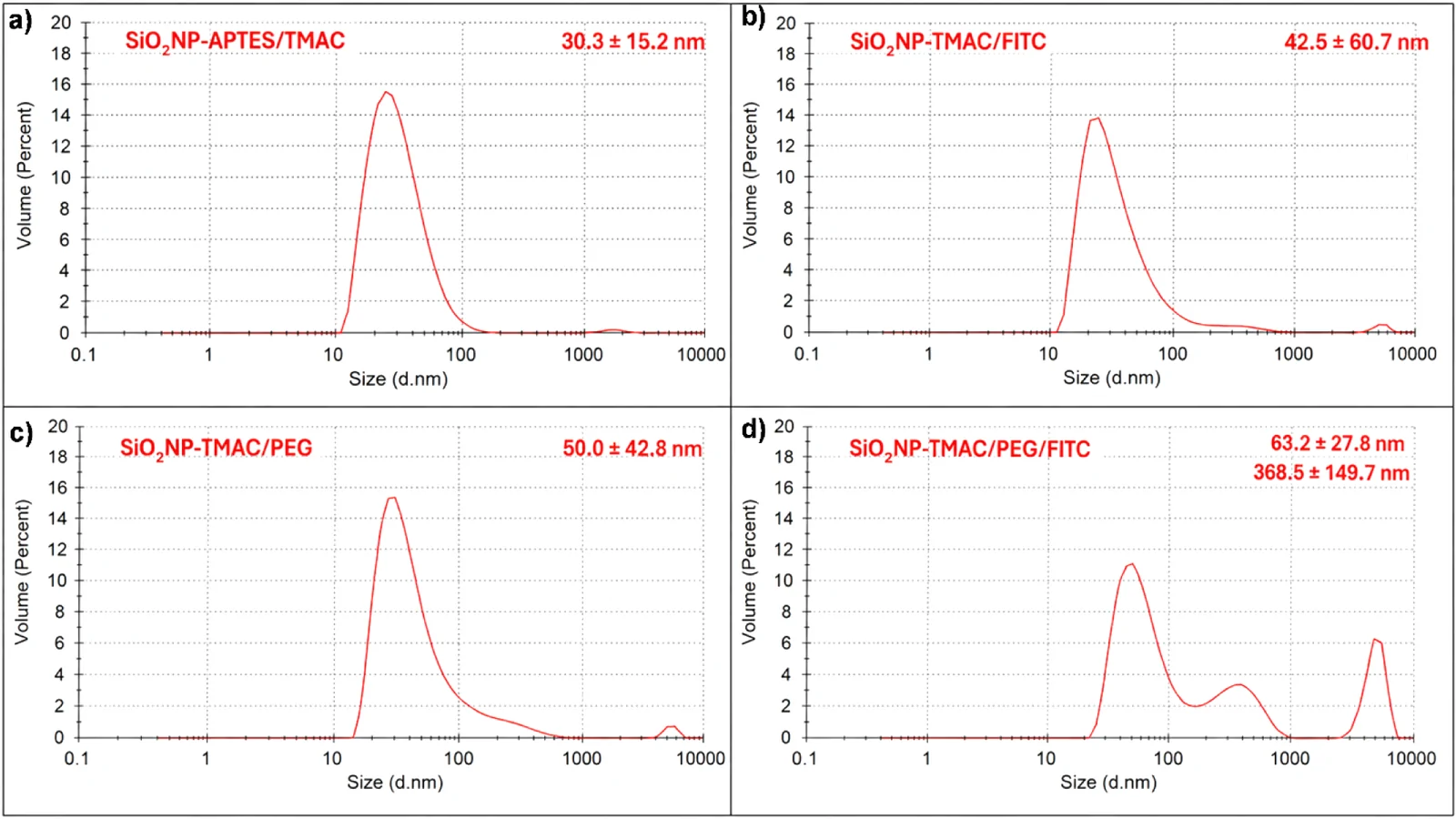
Dynamic light scattering (DLS) size distributions of functionalized silica nanoparticles (SiO_2_NPs): a) dual-functional group SiO_2_NPs (SiO_2_NP-APTMS/TMAC) following conjugation with b) FITC (SiO_2_NP-TMAC/FITC), c) PEG (SiO_2_NP-TMAC/PEG), and d) both FITC and PEG (SiO_2_NP-TMAC/FITC/PEG). The values shown in red indicate the mean hydrodynamic diameter ± standard deviation. The bimodal size distribution observed for SiO_2_NP-TMAC/PEG/FITC is reflected by two particle populations. FITC = fluorescein isothiocyanate; PEG = polyethylene glycol.

**Figure 2:**
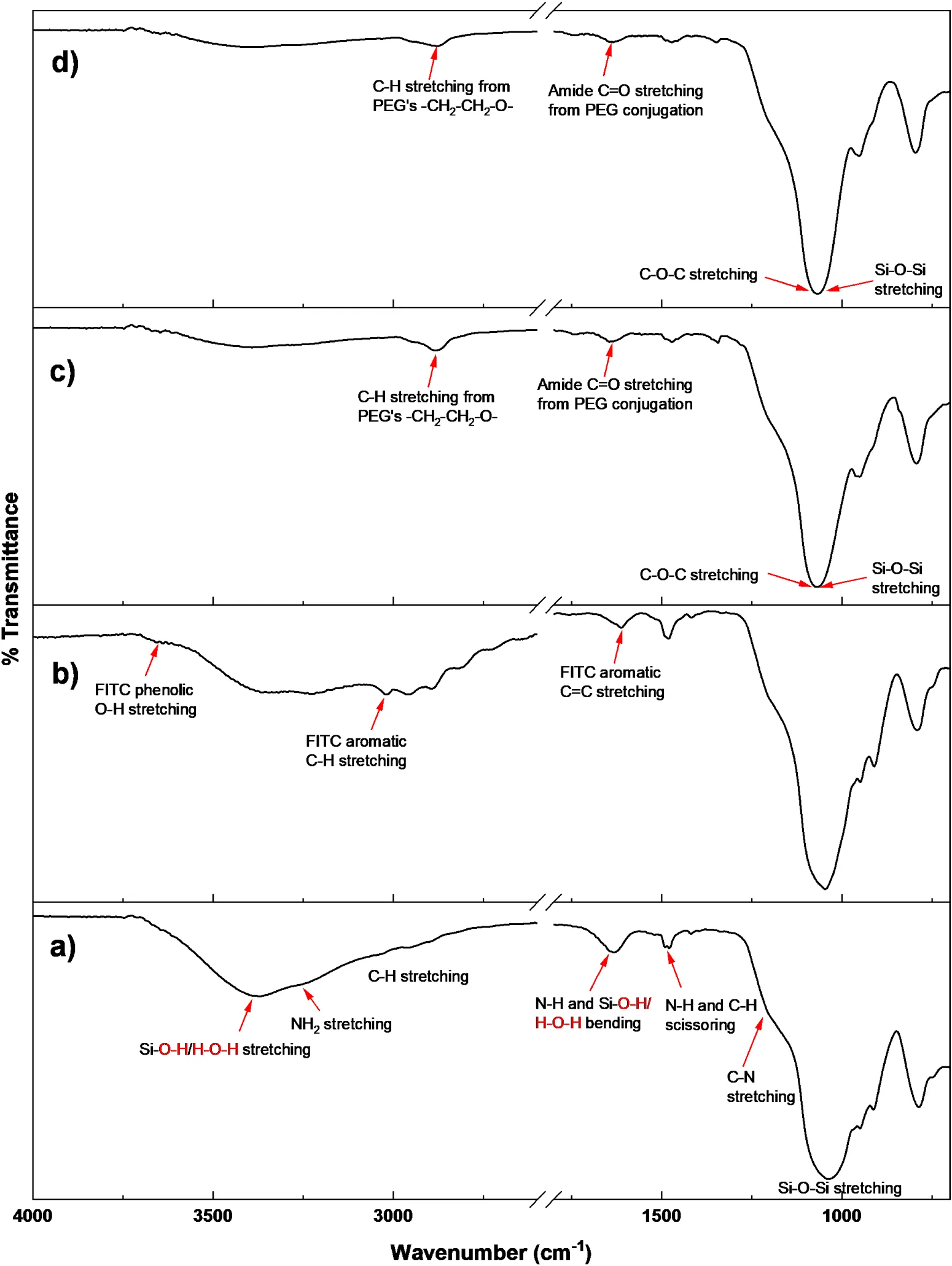
FTIR spectra of a) SiO_2_NP-APTMS/TMAC, b) SiO_2_NP-TMAC/FITC, c) SiO_2_NP-TMAC/PEG, and d) SiO_2_NP-TMAC/PEG/FITC. For panels b, c, and d, only the peaks relevant to the surface modifications have been assigned. FITC = fluorescein isothiocyanate; PEG = polyethylene glycol.

### 2.2 DNA–NP Complexation Efficiency

To evaluate the binding affinity between pDNA and functionalized SiO_2_NPs, a gel retardation assay was conducted using varying DNA:NP (DNA:NP) weight ratios for two representative nanoparticles: the base positively charged SiO_2_NP and the PEGylated, positively charged fluorescent SiO_2_NP. As illustrated in Figure 3, the migration of free pDNA was gradually reduced with increasing NP concentration, indicating progressive complex formation.

**Figure 3:**
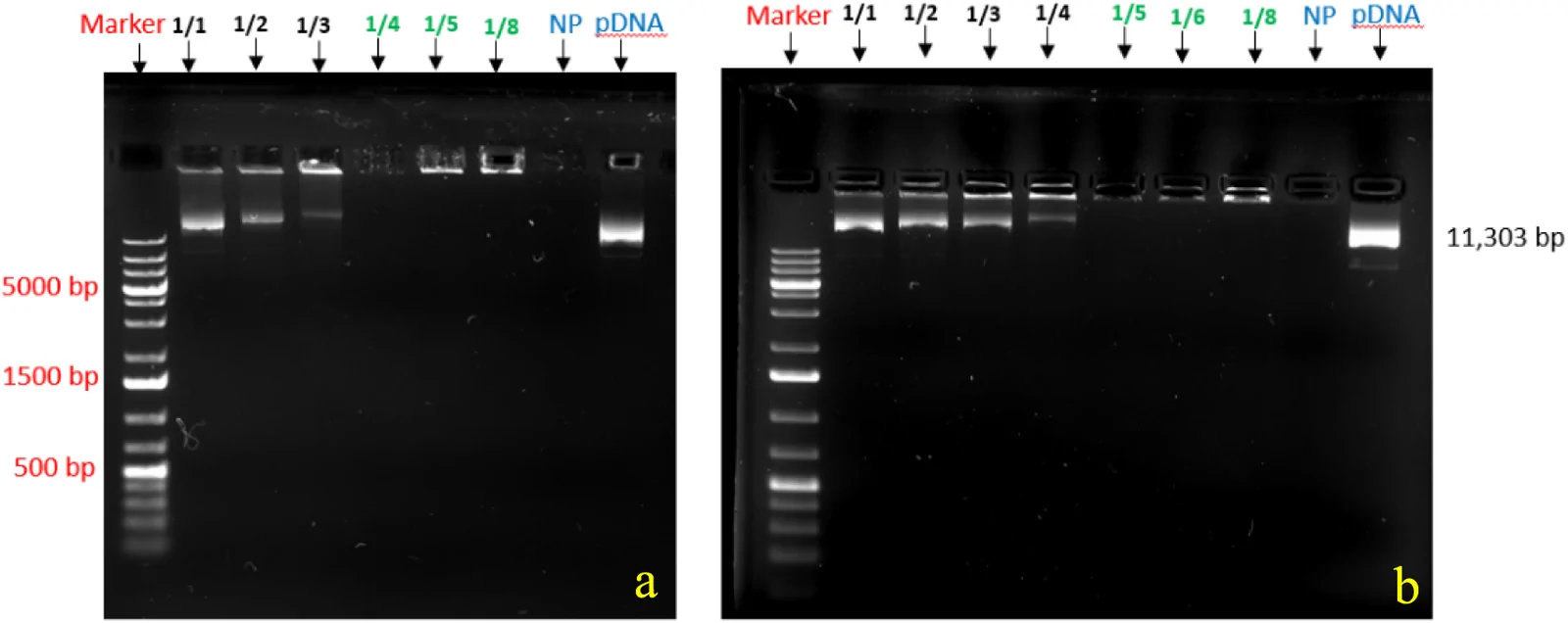
Gel retardation assay showing binding of plasmid DNA (pDNA, 11,303 bp) to functionalized SiO_2_ nanoparticles. Electrophoretic mobility of pDNA mixed with (a) SiO_2_NP-APTMS/TMAC and (b) SiO_2_NP–TMAC/PEG/FITC at increasing DNA:NP weight ratios (1:1, 1:2, 1:3, 1:4, 1:5, 1:6, and 1:8). NP and pDNA denote nanoparticle-only and plasmid-only controls, respectively. Marker: Thermo Scientific GeneRuler 1 kb Plus DNA Ladder (100 bp to 10 kb).

For SiO_2_NP-APTMS/TMAC NPs (without FITC or PEG), complete retardation—evidenced by DNA remaining in the wells—was observed at a 1:4 DNA:NP ratio, suggesting full complexation. In contrast, SiO_2_NP–TMAC/PEG/FITC required a slightly higher ratio, with full DNA retention observed at 1:5, likely due to steric effects and charge shielding introduced by PEG chains that influence binding surface accessibility.^40,41^ The decrease in zeta potential from +45.8 ± 12.4 mV to +27.3 ± 3.5 mV indicates reduced accessible surface charge for SiO_2_NP–TMAC/PEG/FITC. Therefore, the 1:4 and 1:5 ratios were selected as optimal conditions for subsequent transformation experiments involving SiO_2_NP-APTMS/TMAC and SiO_2_NP–TMAC/PEG/FITC, respectively.

### 2.3 Viability Assessment of Canola Protoplasts and Microspores

The viability of isolated canola cells was first confirmed immediately after extraction. Morphological inspection showed that freshly isolated protoplasts (from both hypocotyl and leaf tissues) were predominantly spherical, with smooth membranes and no signs of granulation or lysis (Figure 4a). Fluorescein diacetate (FDA) staining further confirmed their metabolic activity, as viable cells fluoresced green under confocal microscopy (Figure 4b). On average, hypocotyl-derived protoplasts displayed a viability of 87.4 ± 2.6%, while leaf-derived protoplasts showed 83.1 ± 3.1% viability across three biological replicates. Similarly, microspores at the mid- to late-uninucleate stage exhibited strong and uniform FDA-derived green fluorescence, with a mean viability of 78.9 ± 3.7%, confirming intact membranes and active esterase activity before NP treatment.

**Figure 4:**
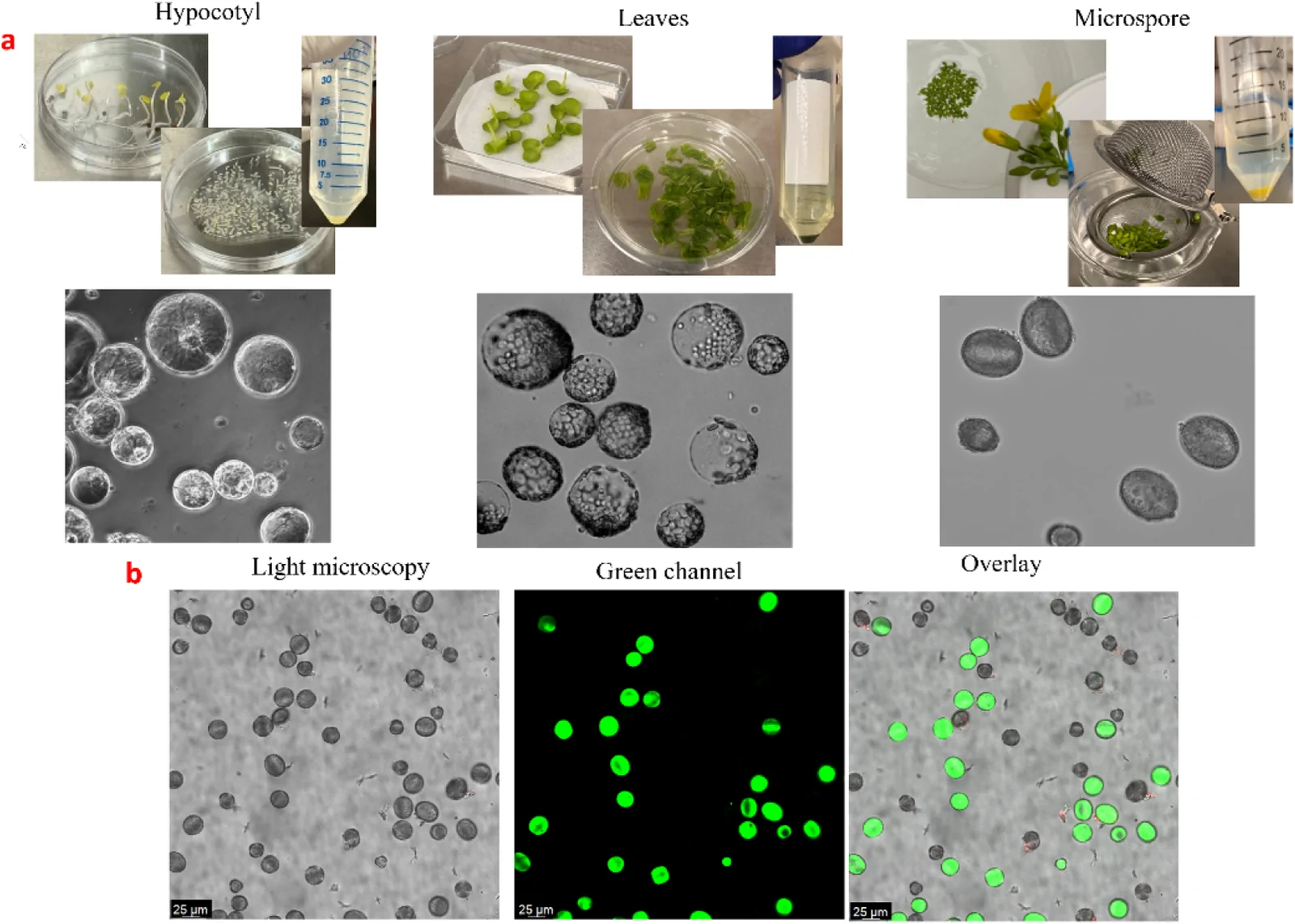
Morphological assessment of isolated canola protoplasts and microspores. a) Representative images illustrating the isolation and morphology of hypocotyl-derived protoplasts, leaf-derived protoplasts, and microspores. b) FDA viability assay of canola microspores showing bright-field, green fluorescence (excitation ∼488 nm/emission ∼520 nm), and merged images. Green fluorescence indicates metabolically active cells. Scale bar = 25 μm.

The cell viability results showed that both hypocotyl- and leaf-derived protoplasts maintained high viability (>80%) at lower concentrations (≤80 ng/mL), but a gradual decline was observed with increasing NP dosage. At the highest concentration tested (200 ng/mL), protoplast viability decreased to ∼65–70%. SiO_2_NP-TMAC/PEG and SiO_2_NP-TMAC/PEG/FITC consistently showed slightly higher viability compared to SiO_2_NPs without PEG (*p* < 0.05), indicating that PEG improved NP dispersibility and reduced toxicity (Figure 5).

**Figure 5:**
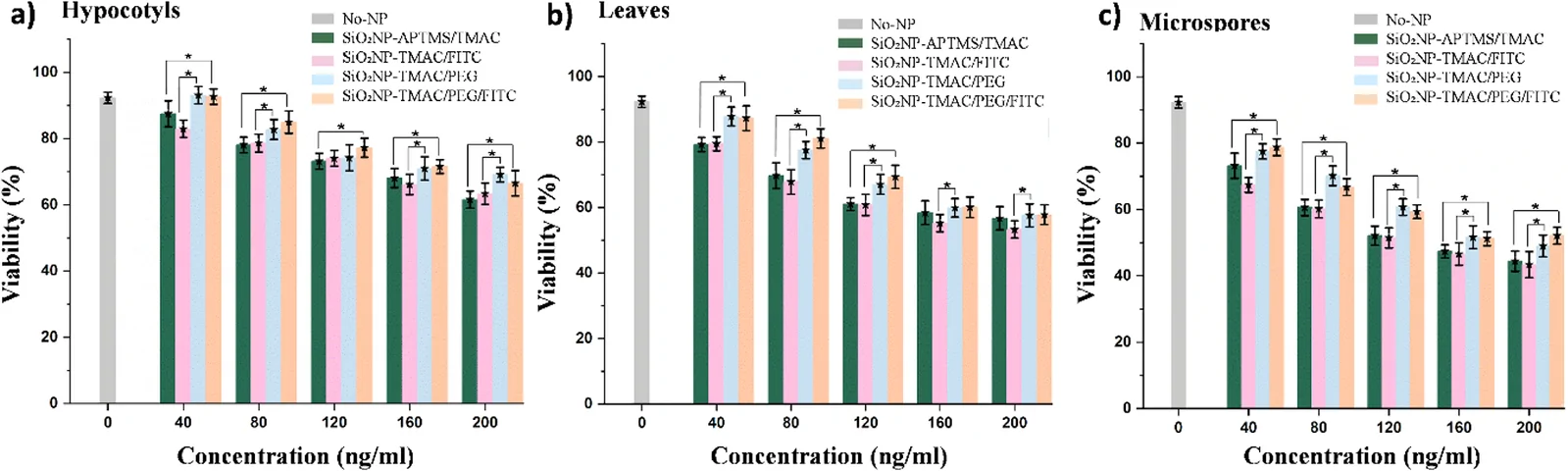
Viability of canola hypocotyl protoplasts (a), leaf protoplasts (b), and microspores (c) following exposure to functionalized silica nanoparticles (SiO_2_NP). Cells were treated with SiO_2_NP-APTMS/TMAC, SiO_2_NP-TMAC/FITC, SiO_2_NP-TMAC/PEG, or SiO_2_NP-TMAC/PEG/FITC and compared with untreated controls (No-NP). Viability was determined by FDA fluorescence and is presented as mean ± SD (*n* = 3). Error bars represent standard deviation. Statistical significance is indicated by *p < 0.05; nonsignificant comparisons are unmarked.

Microspores exhibited greater sensitivity to NP exposure compared to protoplasts. While greater than 75% viability was maintained at NP concentrations up to 80 ng/mL, exposure of microspores to 160–200 ng/mL of NP reduced microspore viability to ∼50–55% across all NP formulations (Figure 5). Again, SiO_2_NP–TMAC/PEG and SiO_2_NP-TMAC/PEG/FITC resulted in significantly higher microspore survival compared to microspores that were exposed to particles without PEG (*p* < 0.05), confirming the protective effect of PEG surface modification. Importantly, morphological inspection of treated microspores showed that cells remained intact and capable of continued division even when microspores were exposed to NPs at concentrations less than 80 ng/mL, whereas higher doses of NPs led to increased membrane irregularities and loss of fluorescence in microspores. Based on these results, the maximum NP concentrations selected for downstream transformation experiments were 80 ng/mL for protoplasts and 60–80 ng/mL for microspores, using NPs with PEG to ensure minimal cytotoxicity.

Additionally, comparing FITC-labeled and unlabeled NPs revealed no statistically significant differences in cell viability across all concentrations tested in both protoplasts and microspores. This confirms that FITC conjugation, used for NP tracking, does not introduce additional cytotoxicity or interfere with particle–cell interactions.

Next, the incubation time was optimized to balance efficient uptake and cell survival. For protoplasts, three time points (1, 4, and 24 h) were evaluated. The 4 h incubation yielded the most consistent internalization and expression with minimal viability loss, while the shorter incubation (1 h) provided insufficient uptake, and longer exposure (24 h) led to reduced viability. For microspores, shorter incubations of 30 and 60 min were tested. The 30 min exposure was optimal, as it allowed efficient NP uptake while maintaining a high cell viability. Longer exposure (60 min) did not improve uptake but led to higher variability in viability, likely because of the increased sensitivity of microspores.

The marked decline in microspore viability at NP concentrations ≥ 160 ng/mL suggests a relatively narrow optimal dose between efficient nanoparticle uptake and cytotoxicity. While lower NP concentrations (≤80 ng/mL) supported substantial cell viability together with successful delivery, higher NP exposure resulted in pronounced reductions in viability, likely due to increased membrane stress and nanoparticle-induced cellular damage. These findings highlight the importance of carefully optimizing NP concentration and exposure conditions for sensitive plant reproductive cells such as microspores and provide practical guidance for future protocol development and nanoparticle-mediated transformation applications. Together, these results demonstrate that both protoplasts and microspores retained substantial viability after NP exposure, and the SiO_2_NPs with PEG at optimized concentrations (≤80 ng/mL) provided the best balance of uptake efficiency and cytocompatibility. These optimized conditions were therefore used in subsequent transformation experiments.

### 2.4 Analysis of pDNA/Functionalized SiO_2_NP Complex Delivery in Canola Protoplasts and Microspores

In protoplast transformation experiments, we compared cells isolated from both leaf and hypocotyl tissues under identical conditions and at equal cell densities. Hypocotyl-derived protoplasts consistently exhibited higher transformation efficiency and more sustained transgene expression compared with leaf-derived protoplasts. Based on these observations, hypocotyl protoplasts were selected for subsequent analyses.

Fluorescence microscopy confirmed successful uptake of DNA–NP complexes in both protoplasts and microspores (Figures 6 and 7), as indicated by green FITC signals from the SiO_2_NPs and red signals from the reporter gene, providing dual confirmation of nanoparticle internalization. In protoplasts, transformation events were readily detected across all four variations of functional SiO_2_NP treatments, with notably higher frequencies observed when SiO_2_NPs with PEG were used. Quantification showed that PEG-modified particles achieved transformation efficiencies of approximately 20–25%, compared with 10–12% for SiO_2_NPs without PEG modification. In microspores, transformation frequencies were lower overall, yet a consistent increase was observed with PEG-modified silica NPs (8–10%) compared to non-PEG NPs (4–5%). These data indicate that PEG surface modification enhances the delivery capacity of SiO_2_NPs in both cell types, although the absolute efficiency was higher in protoplasts than in microspores.

**Figure 6:**
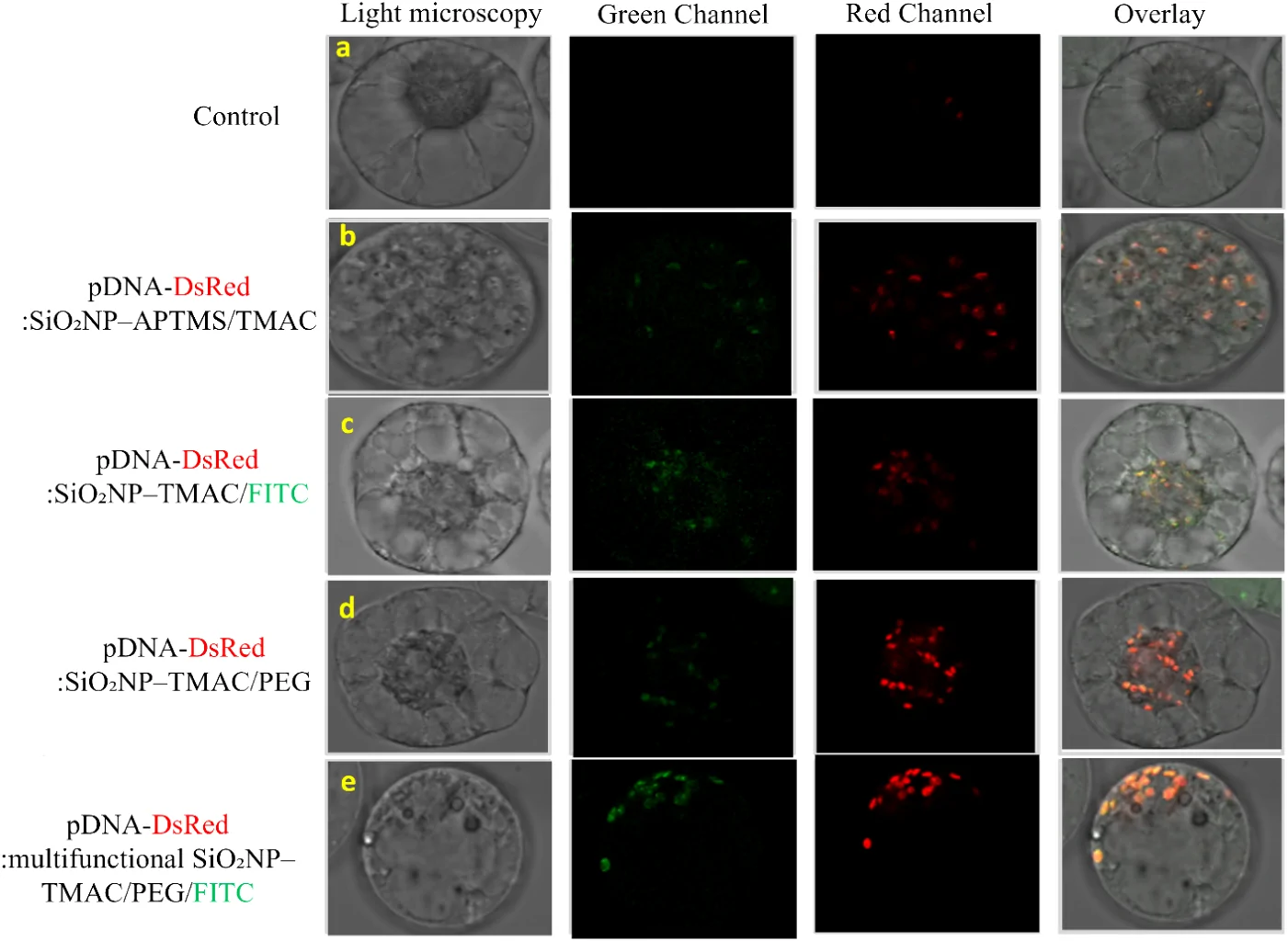
Confocal microscopic analysis of pDNA:SiO_2_NP complex uptake and DsRed expression in canola hypocotyl-derived protoplasts. Representative images show bright-field, FITC fluorescence (excitation ∼488 nm/emission ∼520 nm), DsRed fluorescence (excitation ∼558 nm/emission ∼583 nm), and merged overlays. Treatment groups include a) untreated control, b) pDNA:SiO_2_NP-APTMS/TMAC, c) pDNA:SiO_2_NP–TMAC/FITC, d) pDNA:SiO_2_NP–TMAC/PEG, and e) pDNA:SiO_2_NP–TMAC/PEG/FITC. Green fluorescence corresponds FITC-labeled nanoparticles, whereas red fluorescence corresponds to DsRed reporter expression. Images were acquired 48 h after treatment.

**Figure 7:**
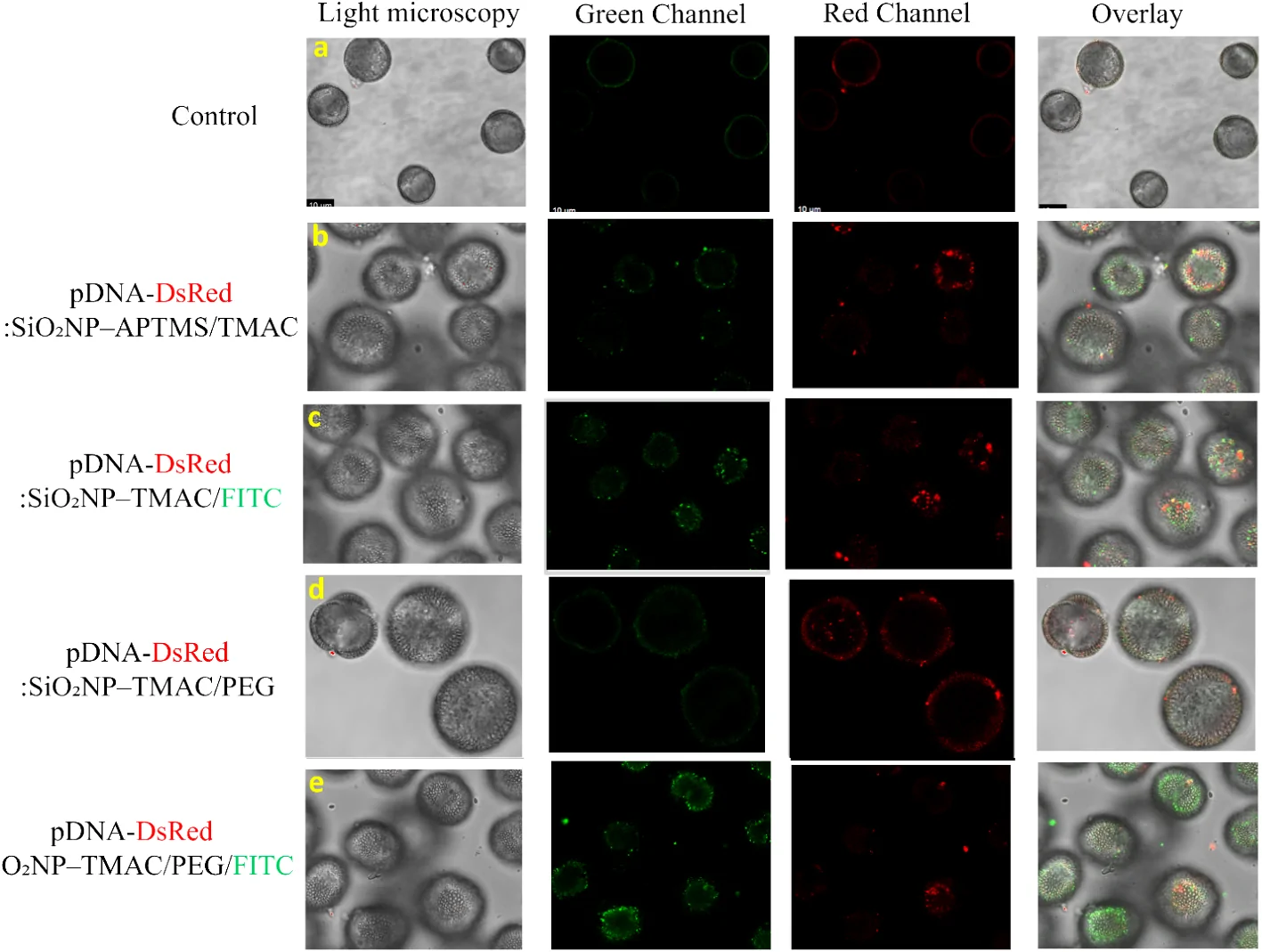
Confocal microscopic analysis of pDNA:SiO_2_NP complex uptake and DsRed expression in canola microspores. Representative images show bright-field, FITC fluorescence (excitation ∼488 nm/emission ∼520 nm), DsRed fluorescence (excitation ∼558 nm/emission ∼583 nm), and merged overlays. Treatment groups include a) untreated control, b) pDNA:SiO_2_NP-APTMS/TMAC, c) pDNA:SiO_2_NP–TMAC/FITC, d) pDNA:SiO_2_NP–TMAC/PEG, and e) pDNA:SiO_2_NP–TMAC/PEG/FITC. Green fluorescence corresponds FITC-labeled nanoparticles, whereas red fluorescence corresponds to DsRed reporter expression. Images were acquired 24 h after treatment.

Microspore preparations yield cell populations of varying sizes, and we hypothesized that these size differences influence responses to NP treatment. When comparing the regeneration rates (Figure 8) of two sizes of microspores (1–2 mm and 3–4 mm) at 1 week after transformation (WAT), an interesting difference was observed. In the control (no NP treatment), smaller microspores (1–2 mm) exhibited a relatively higher regeneration rate. However, in the NP-treated samples, larger microspores (3–4 mm) showed superior regeneration efficiency and more vigorous growth. Based on these results, 3–4 mm microspores were selected for subsequent experiments. Although control microspores generally exhibited more vigorous growth than NP-treated samples during later developmental stages, regenerated plantlets were successfully obtained from both NP-treated groups, indicating that nanoparticle exposure did not prevent regeneration and plant development (Figure 8).

**Figure 8:**
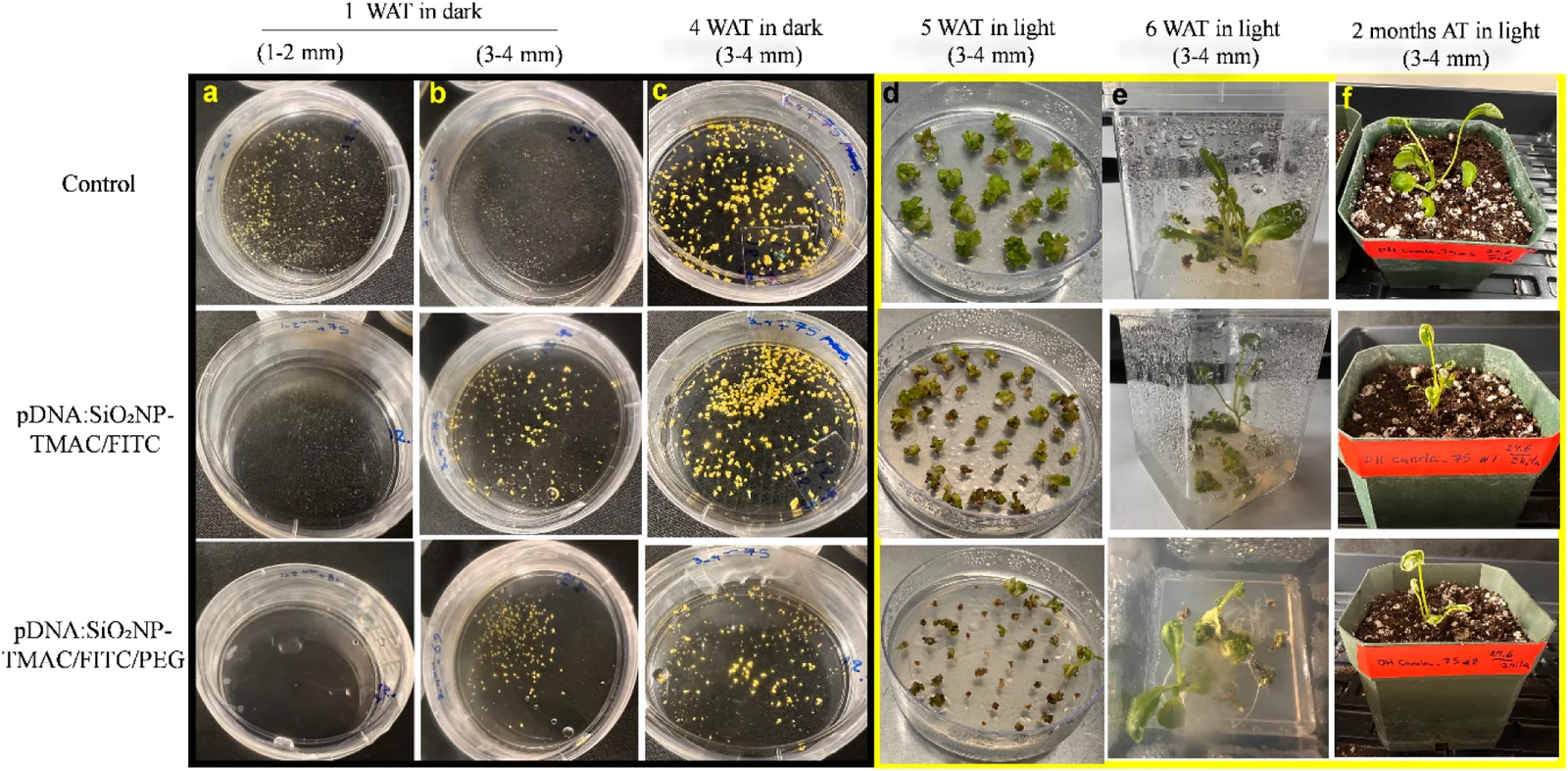
Regeneration of canola microspores following transformation with DNA-DsRed/SiO_2_ NP complexes. The top row shows untreated control microspores, whereas the middle and bottom rows show microspores treated with pDNA: SiO_2_NP-TMAC/FITC and pDNA: SiO_2_NP-TMAC/PEG/FITC, respectively. Panels (a) and (b) show microspores derived from 1–2 mm and 3–4 mm flower buds, respectively, at 1 week after transformation (WAT) under dark conditions. Panel (c) shows microspores at 4 WAT in liquid medium under dark conditions. Panels (d) and (e) show regeneration and plant development at 5 and 6 WAT under light conditions, respectively. Panel (f) shows regenerated plantlets after 2 months of growth in soil under light conditions. Black borders indicate dark incubation, and yellow borders indicate light exposure.

To further evaluate transgene delivery in regenerated plants, genomic DNA was extracted from four regenerated canola plantlets and analyzed by PCR using DsRed-specific primers (Figure 9). A PCR product of the expected size (678 bp) was detected in one regenerated plant (line 2), while no amplification was observed in the wild-type control. The plasmid DNA used for transformation served as a positive control and yielded an expected amplicon. These results provide molecular evidence for the presence of the delivered DsRed transgene in at least one regenerated plant obtained by following nanoparticle-mediated gene delivery.

**Figure 9:**
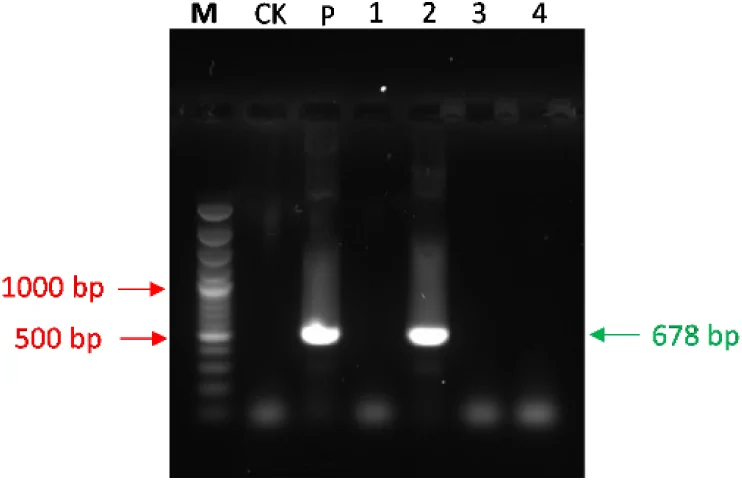
PCR analysis of the DsRed transgene in regenerated canola plants. Genomic DNA extracted from four regenerated canola plantlets was amplified using DsRed-specific primers, yielding an expected amplicon size of 678 bp. Lane M: Thermo Scientific GeneRuler 100 bp Plus DNA Ladder; CK: wild-type control plant; P: pBinGlyRed3 plasmid (positive control); lanes 1–4: regenerated canola plantlets.

## 3 Discussion

In this work, we have demonstrated a novel nanotechnology-based method for delivering genetic materials into *B. napus* using surface-modified SiO_2_ NPs. Dual-functional group modification endowed NPs with complementary properties: the quaternary ammonium groups enabled strong electrostatic binding with negatively charged DNA, while the primary amine groups provided sites for covalent attachment of fluorescent dyes (e.g., FITC) and/or PEG. This combination not only supported DNA complexation but also enhanced tracking and facilitated cellular uptake.

In particular, PEG conjugation is known to increase membrane permeability by destabilizing the plasma membrane, thereby promoting endocytosis—a process often referred to as PEG-mediated transformation.^42^ The strongly positive surface charge derived from cationic quaternary ammonium groups is expected to enhance interactions with the negatively charged outer layers of cell membranes, improving internalization efficiency.^43^

The improved dispersibility of functionalized particles in water, together with their homogeneous distribution observed in SEM, suggests effective stabilization against aggregation. Spectroscopic analyses (NMR and XPS) confirmed a higher proportion of quaternary ammonium groups relative to primary amines, ensuring that even when primary amines are consumed during covalent conjugation, the NPs retain a strong overall positive charge. TGA revealed ∼14% organic content, consistent with sufficient incorporation of surface functional groups.^39^ Overall, the dual-functional group strategy yields colloidally stable NPs with essential features for DNA binding, covalent modification, membrane interaction, and efficient cellular delivery. Coupling of FITC dye to SiO_2_NP-APTMS/TMAC via thiourea bond formation yielded yellow-colored particles with green fluorescence. The fluorescence allowed tracking of the nanocarriers during cellular internalization, while the stable thiourea linkage provided hydrolytic stability under DNA transformation conditions. The hydrodynamic diameter showed no significant increase, and the ζ-potential remained strongly positive, both favorable for cell entry. The high positive charge also promoted efficient anchoring of negatively charged DNA, although excessively high surface charge may contribute to dose-dependent cytotoxicity. PEG functionalization via amide coupling with surface amines might have partially screened the positive charge, reducing potential toxicity while enhancing DNA transformation efficiency, as PEG is known to promote protoplast uptake through endocytosis.^42^ The hydrodynamic diameter nearly tripled compared to SiO_2_NP-APTMS/TMAC, which could be beneficial for endocytosis-mediated internalization. Dual conjugation with both FITC and PEG via thiourea and amide bonds further increased particle size more than 3-fold and produced a higher fraction of aggregates due to the additional hydrophobicity introduced by FITC. These modifications collectively are supposed to favor endocytosis, with PEG mitigating charge-related toxicity and enhancing delivery, while FITC enabled fluorescent tracking of the DNA–NP complexes.

PEG-modified silica NPs consistently outperformed other functional SiO_2_NPs, leading to higher transformation efficiencies and improved viability in both cell types. In protoplasts, PEG-modified silica NPs achieved transformation efficiencies of ∼20–25%, compared to 10–12% for non-PEG SiO_2_NPs. In microspores, the overall rates were lower (8–10% vs 4–5%) but showed the same improvement trend. The superior performance of PEG-modified silica NPs is likely explained by two factors: first, PEG promotes pore formation and endocytic uptake; second, PEG steric shielding, together with the lower zeta potential of the PEG-functionalized silica nanocarrier, might have enabled reversible DNA binding. This might have allowed faster release of DNA from endosomes into the cytosol, in contrast to SiO_2_NP–APTMS/TMAC, which has a much higher positive charge and no PEG shielding, leading to stronger irreversible binding and reduced release. Moreover, PEG improved colloidal stability under highly saline buffered conditions and reduced nonspecific interactions, thereby facilitating efficient uptake, intracellular trafficking, and lowering cytotoxicity.^33,34,44^ Our observations align with prior studies reporting that surface modifications with PEG improve NP dispersion and reduce cytotoxicity.^44^ Importantly, this increased efficiency was achieved while maintaining high cell viability, highlighting PEGylation combined with positively charged quaternary ammonium groups as a key strategy for optimizing SiO_2_NPs in plant transformation. Meanwhile, green FITC conjugation helped visualize nanocarrier uptake into the cell, along with red fluorescence from the DsRed DNA reporter gene, providing dual confirmation of DNA–NP complex internalization.

While PEGylation likely contributed to improved nanoparticle uptake and gene delivery efficiency, the precise mechanism by which the multifunctional SiO_2_ nanoparticles traverse the highly resistant microspore exine and intine layers remains unclear. Previous studies have reported that the effective size exclusion limit of plant cell walls is often in the range of 20–50 nm, although this value varies depending on plant species, cell type, and wall architecture.^45,46^ Nevertheless, nanoparticles larger than the nominal pore size have also been reported to enter plant cells through interactions that modify local wall permeability and facilitate transport across the cell wall barrier.^47,48^ Given that the multifunctional SiO_2_ nanoparticles used in this study exhibited a hydrodynamic diameter of approximately 63 nm, uptake may have occurred through naturally occurring aperture regions and/or localized variations in wall permeability, potentially facilitated by the presence of cationic charges and the temporary membrane/wall-destabilizing effect of PEG bonded to the SiO_2_ nanoparticles. However, the transport pathway was not directly investigated in the present study. Therefore, further studies employing high-resolution imaging techniques will be required to elucidate the exact mechanism of nanoparticle entry into canola microspores.

Another important observation was the temporal stabilization of gene expression in protoplasts following the NP treatment. Red fluorescence signals from reporter gene DsRed-DNA appeared shortly after NP treatment, peaked, and stabilized by 48 h, consistent with earlier reports on transient expression in plant protoplast systems.^29^ This suggests that most NP uptake and DNA release occur within the first 2 days, making 48 h a reliable time point for evaluating transformation outcomes across tissues.

Microspore preparations yield cell populations of varying sizes, and we hypothesized that these size differences may influence responses to NP treatment. A size-dependent response of microspores to NP treatment was evident when comparing regeneration outcomes. In untreated controls, smaller microspores (1–2 mm buds) displayed higher regenerative capacity, consistent with their earlier developmental stage and inherently greater embryogenic potential. However, following functionalized SiO_2_NP exposure, larger microspores (3–4 mm buds) demonstrated enhanced regeneration efficiency and more robust growth responses. This shift suggests that, while smaller microspores are intrinsically more totipotent, larger microspores are more permissive to NP uptake due to increased cytoplasmic volume and surface area, resulting in improved tolerance and functional response to NP-mediated delivery.

The relationship between cell size and transformation responsiveness was not limited to microspores, as a similar pattern was observed in protoplast experiments, where hypocotyl-derived protoplasts—which are typically larger and more homogeneous than leaf-derived ones—exhibited higher transformation efficiency and more stable gene expression. This consistency across two distinct cell systems suggests that cell size and uniformity are key determinants of nanoparticle–cell interactions and subsequent DNA uptake efficiency. The difference in protoplast performance may be explained by the larger and more homogeneous size distribution of hypocotyl protoplasts, which provides greater surface area for NP interaction and uptake, thereby increasing the likelihood of DNA delivery events.^37,49^ Supporting evidence comes from carrot, where hypocotyl protoplasts were found to be significantly larger than leaf-derived ones and displayed better plating efficiency and plasmolysis resilience.^50^ Similarly, in cabbage, hypocotyl protoplasts showed superior regeneration capacity.^51^ Rouan et al. further demonstrated a direct correlation between protoplast size and the critical field strength required for electropermeabilization, highlighting that larger cells are more amenable to molecular uptake.^52^ Although their study involved electroporation, the same principle applies to NP-mediated transformation, where the larger surface area and membrane accessibility promote NP binding and internalization. These precedents support our finding that hypocotyl-derived protoplasts provide a more favorable system for NP-mediated transformation in *B. napus*.

Differences in transformation between protoplasts and microspores highlight cell-type-specific responses. Protoplasts, which lack cell walls, were more accessible to NPs, whereas microspores—despite their embryogenic potential—showed lower efficiency, likely due to structural barriers and higher physiological sensitivity. Within microspores, the developmental stage strongly influenced responsiveness: smaller microspores (from 1 to 2 mm buds) showed higher regeneration in controls, consistent with their higher totipotency,^11,44^ whereas NP-treated cultures regenerated better with larger microspores (from 3 to 4 mm buds). Larger cells may provide greater cytoplasmic volume and surface area, dilute cytotoxic effects, and ensure more uniform NP uptake.^49,51,53^ Thus, optimizing the microspore stage is critical for balancing embryogenic potential with NP-mediated delivery efficiency.

Microscopy confirmed colocalization of SiO_2_NP–TMAC/FITC and SiO_2_NP–TMAC/FITC/PEG (green) with DsRed fluorescence reporting successful transgene expression (red) in both protoplasts and microspores. In protoplasts, signals persisted for up to 72 h, with DsRed expression continuing for up to a week. FITC fluorescence near or surrounding the nucleus suggests nuclear access of the DNA–NP complexes, whereas DsRed fluorescence confirms successful transcription and translation of the delivered gene but does not directly indicate nuclear localization of the protein. The ability of transformed protoplasts to remain viable, divide, and form microcalli underscores the regeneration potential of this system. However, confirming genomic integration and heritable expression requires further work, particularly the regeneration of whole plants and molecular validation. Achieving this would necessitate the design of targeted constructs that deliver genes of interest directly into the genome. In this context, genome editing strategies such as CRISPR/Cas could be combined with NP-mediated delivery to promote precise integration and stable transformation.^37,54^

Our findings also align with those of an earlier work using alternative nanocarriers. For instance, rod-shaped nanostructures (RNTs) were used in wheat microspores to deliver DNA, but uptake was limited by the strong negative charge of free DNA, and red fluorescence was largely cytoplasmic, suggesting that nuclear delivery was inefficient.^55^ In contrast, PEGylated positively charged FITC-labeled SiO_2_NPs in this study showed efficient intracellular uptake in both canola protoplasts and microspores, with FITC fluorescence frequently observed near the nucleus and DsRed expression confirming the functional delivery of the transgene. These outcomes indicate that PEG surface modification enhances nanoparticle dispersion, minimizes nonspecific interactions, and may facilitate more effective transport toward the nucleus. While differences in nanoparticle type, plant species, and cellular architecture may also contribute to the observed improvements, these results collectively highlight the potential of PEGylated, positively charged SiO_2_NPs to overcome major cellular barriers to gene delivery. Efficient uptake, proximity of nanoparticles to the nucleus, and sustained cell viability may therefore represent key determinants for advancing future nanoparticle-mediated transformation strategies in *B. napus*. Comprehensive comparisons of nanoparticle-mediated plant gene delivery systems, including nanoparticle characteristics, delivery approaches, cytotoxicity, transformation efficiency, and regeneration-related challenges, have been summarized in our recent reviews.^17,32^ Compared with previously reported nanoparticle systems, the PEGylated, positively charged fluorescent SiO_2_NP platform developed here integrates efficient plasmid DNA binding, improved cytocompatibility, and fluorescence-based tracking within a single nanocarrier. In addition, it was successfully applied to both wall-free protoplasts and cell-walled microspores of *B. napus*, enabling the direct evaluation of DNA delivery, cell viability, and regeneration potential in the same crop system.

Surface modification of SiO_2_NPs contributed to improved DNA uptake and cell viability in both protoplasts and microspores, consistent with the enhanced intracellular transport observed in the current study. In this delivery platform, the SiO_2_NPs core provides structural stability and high surface area for DNA association, while the surface modification of SiO_2_NPs improves NP dispersibility, reduces nonspecific interactions, and minimizes cytotoxicity by potentially masking excessive surface charge. These positive charges are attracted to the negatively charged cell membrane, facilitating internalization. In addition, PEG enhances uptake by destabilizing the cell membrane, working together with the NP surface charges. This cumulative effect of PEG and positive charges might have also destabilized the endosomal membrane, allowing the DNA–NP complex to escape into the cytosol and avoid lysosomal trafficking, where enzymatic DNA degradation occurs.^56,57^ The high positive charges on quaternary ammonium-functionalized SiO_2_NPs (e.g., SiO_2_NP–APTMS/TMAC) promote strong complex formation with DNA, which could hinder its release into the cytosol before nuclear entry. In contrast, PEGylation of SiO_2_NPs screens surface charge and provides steric hindrance, presumably weakening the complex strength and thereby facilitating DNA release into the cytosol and subsequent nuclear entry. In line with this hypothesis, Thomas and Klibanov highlighted that appropriate DNA condensation within NPs is a prerequisite for achieving high transfer efficiency, which is consistent with our observations.^58^ The relatively low cytotoxicity, simplicity, and efficiency of PEGylated, positively charged, FITC-labeled SiO_2_NPs therefore make them attractive alternatives to conventional DNA delivery methods in plant systems. Moreover, when combined with precise genome editing tools such as CRISPR/Cas, NP-mediated DNA delivery could serve as a foundation for robust and targeted genome engineering protocols in plants.

PCR analysis confirmed the presence of the DsRed transgene in the regenerated canola plant, providing preliminary molecular evidence that nanoparticle-mediated delivery can result in the recovery of transgene-positive regenerated plants. However, additional analyses, including transgene copy number determination, integration site characterization, and inheritance studies, will be required to fully assess stable transformation and genomic integration. Overall, PEGylated positively charged FITC-labeled SiO_2_NPs demonstrate strong potential as a platform for NP-mediated transformation in *B. napus*. NP systems provide unique benefits: independence from genotype constraints, nonintegrative delivery, and compatibility with gene-editing tools such as CRISPR/Cas.^35^ Global regulatory frameworks governing genetically modified organisms (GMOs) have increasingly motivated the development of alternative plant gene delivery strategies that avoid stable genomic integration. In this context, transient gene expression systems, in which the delivered genetic material is not permanently incorporated into the plant genome, are gaining considerable attention. Such approaches enable functional validation or genome editing while minimizing regulatory and biosafety concerns associated with transgenic plants. For example, polymer- and carbon nanotube-based nanocarriers have been successfully used to deliver DNA or RNA into plant tissues, resulting in gene-edited seedlings without stable transgene integration.^26,59–61^ Similarly, direct delivery of CRISPR–Cas9 ribonucleoprotein complexes into plant cells, including canola, has enabled precise genome editing without leaving a transgenic footprint. Future work should build on these findings by refining NP properties, optimizing cell type and developmental stage selection, and combining this approach with traditional transformation methods to enhance stable integration and improve efficiency in canola and other crops.

## 4 Conclusion

This study demonstrated the potential of PEGylated positively charged fluorescent SiO_2_NPs as efficient carriers for DNA delivery into *B. napus* protoplasts and microspores. Optimization of NP concentration, incubation time, and cell source revealed that hypocotyl-derived protoplasts provided higher transformation efficiency and more consistent outcomes compared with leaf-derived protoplasts. Surface modification of SiO_2_NPs with PEG, together with the primary DNA-binding positively charged quaternary ammonium groups, played a critical role in enhancing nanoparticle stability, reducing cytotoxicity, improving DNA uptake across cell types, and potentially facilitating efficient cytosolic release and nuclear entry of the DNA. Meanwhile, FITC conjugation enabled green-channel fluorescence tracking of DNA–NP internalization. This, combined with red fluorescence from the DsRed DNA reporter gene, provided complementary confirmation of DNA–NP uptake. Microspore developmental stage influenced responsiveness, with larger microspores performing better following NP treatment. Collectively, these findings highlight the potential of PEGylated positively charged fluorescent SiO_2_NPs–mediated DNA delivery as a flexible and genotype-independent approach. Future studies should build on this work by refining NP properties and exploring their integration with established transformation platforms such as Agrobacterium- or biolistic-mediated methods to further enhance transformation outcomes in canola and related crops. We are currently extending this work toward PEGylated positively charged fluorescent SiO_2_NPs–mediated delivery approaches that could enable gene silencing or targeted gene expression in *B. napus*.

Although the present study successfully demonstrated transient gene delivery and expression in canola protoplasts and microspores, future studies are required to molecularly validate genomic integration and regeneration of stable transgenic lines. Such investigations will be important for evaluating the long-term applicability of this nanoparticle-mediated delivery platform to plant biotechnology and crop improvement.

## 5 Experimental Procedures

### 5.1 Plant Material and Growth Conditions

Seeds of *B. napus* cv. Westar were obtained from the University of Saskatchewan, Saskatoon, Saskatchewan, Canada. The seeds were surface-sterilized with 70% ethanol for 1 min, followed by rinsing 3–4 times with sterile distilled water. For further sterilization, seeds were soaked for 30 min in a solution containing 20% (v/v) commercial bleach (5% NaOCl) and 0.1% (v/v) Tween-20 under gentle stirring, then rinsed thoroughly. The sterilized seeds were germinated in glass jars containing solid MS medium (4.33 g/L) supplemented with 7 g/L bacto-agar, adjusted to pH 5.8, and incubated in the dark at 22 °C. For microspore culture, healthy and uniform *B. napus* cv. Westar plants were grown under controlled greenhouse conditions (16 h light/8 h dark photoperiod, 22 ± 2 °C, and 60% relative humidity).

### 5.2 Protoplast Isolation

Protoplast isolation was performed following the protocol of Li et al. with minor modifications.^2^ Briefly, protoplasts were extracted from hypocotyls of 4–5-day-old seedlings and leaves of 3–4-week-old seedlings. Plant tissue was finely chopped and preincubated in plasmolysis solution (0.4 M mannitol, pH 5.7) for 30–60 min in the dark. Enzymatic digestion was then carried out in a solution containing 1.5% cellulase (Onozuka R-10, Fisher Scientific, USA), 0.6% macerozyme R10 (Fisher Scientific, USA), 0.1% BSA, 1 mM CaCl_2_, 1 mM β-mercaptoethanol, 10 mM MES, and 0.4 M mannitol (pH 5.7). The solution was prewarmed to 55 °C for 10 min to inactivate proteases, cooled, and filter sterilized. Tissues were digested overnight (14–16 h) at room temperature in the dark with gentle shaking (50 rpm).

The resulting protoplast suspension was filtered through a 70 μm mesh (hypocotyls) or 40 μm mesh (leaves), then centrifuged at 100 × *g* for 10 min. The protoplast pellet was washed three times with freshly prepared W5 solution (154 mM NaCl, 125 mM CaCl_2_, 5 mM KCl, 2 mM MES, pH 5.7). Finally, the cell density was adjusted to 4–6 × 10^5^ cells/mL in 0.5 M mannitol for transformation.

### 5.3 Microspore Isolation and Culture

Young inflorescences were harvested, and unopened buds (1–2 mm and 3–4 mm) were selected. Buds were surface sterilized in 3% bleach for 10 min with intermittent shaking, then rinsed three times with sterile distilled water. The sterilized buds were transferred to a sterile 20 mL glass beaker and gently crushed with a sterile glass rod in 2 mL of cold B5–13 liquid medium prepared from Gamborg’s B5 basal medium powder (3.21 g/L; Sigma-Aldrich, USA) supplemented with 13% (w/v) sucrose and adjusted to pH 6.0. Microspores were released by applying gentle pressure with a sterile plunger. The suspension was filtered through a 40 μm nylon mesh and subjected to three rounds of centrifugation at 130 × *g* for 3 min at 4 °C, with each round followed by resuspension in fresh cold B5–13 medium. After the final centrifugation, the pellet was resuspended in NLN-13 medium prepared from NLN basal salts (1.4 g/L; Fisher Scientific, USA) supplemented with 13% (w/v) sucrose and 3.38 mM Ca­(NO_3_)_2_·4H_2_O, adjusted to pH 6.0. Microspore density was then adjusted to 4 × 10^4^ cells/mL using a hemocytometer.

To induce embryogenesis, Petri dishes containing microspores in NLN-13 medium were incubated at 32 °C in darkness for 3 days (heat shock), followed by incubation at 25 °C in darkness. Embryo development was monitored microscopically until structures reached 1–5 mm in size. Developing embryos were then transferred to half-strength B5 solid medium and stored at 4 °C for 4 weeks. Embryos that turned green were subsequently cultured under light conditions to promote the formation of shoots and roots.

### 5.4 Protoplast and Microspore Viability

Protoplast viability was assessed using trypan blue exclusion staining. A 10 μL aliquot of the isolated protoplast suspension was mixed with 10 μL of 0.4% trypan blue and loaded onto a hemocytometer for analysis under a light microscope. The percentage of viable (clear) cells was calculated using the following formulas:

Total cells/ml=(Total cells counted×Dilution factor)/number of
squares×10000

Viable cells(%)=[Total number of viable cells (unstained)/Total number of viable (clear) and nonviable (blue)
cells]×100

In our previous study,^18^ we reported that Trypan Blue formed visible aggregates when protoplasts were suspended in W5 or MMG buffer. This was attributed to the presence of Ca^2+^ and Mg^2+^ ions, which may bind to the sulfonate groups of the dye, reducing its solubility and interfering with cell counting. To address this, CaCl_2_ and MgCl_2_ were omitted from W5 and MMG buffers during Trypan Blue staining in the current study.

Additionally, protoplast and microspore viability was evaluated using FDA staining. A 0.2% FDA stock solution was first diluted 1:10 in W5 (for protoplasts) or NLN-13 (for microspores) and then mixed with the respective cell suspensions. The mixtures were incubated at 4 °C in the dark for 10 min. To evaluate cytocompatibility with functionalized SiO_2_NPs, protoplast and microspore suspensions were incubated with increasing concentrations (40–200 ng/mL) of SiO_2_NPs in different formulations (SiO_2_NP-APTMS/TMAC, SiO_2_NP-TMAC/FITC, SiO_2_NP-TMAC/PEG, and SiO_2_NP-TMAC/PEG/FITC). Cell viability was quantified using FDA staining and analyzed with GraphPad Prism version 9.5.1 (Figure 5). Viability was calculated as the percentage of FDA-positive (green fluorescent) cells relative to the untreated control, which was defined as 100% viability for each corresponding cell type. After which, viability was assessed by fluorescence microscopy using a confocal laser scanning microscope. Viable cells fluoresced green due to esterase activity in intact, metabolically active cells. To determine the optimal NP concentration for transformation, viability was assessed both before and after incubation with silica NPs. Morphological criteria (e.g., cell shape and membrane integrity) were combined with trypan blue and FDA staining to evaluate cytocompatibility.

### 5.5 Surface-Tailored Solid Silica NPs: Synthesis and Characterization

Four types of SiO_2_NPs were evaluated for gene delivery: (1) SiO_2_NP–NH_2_/N­(CH_3_)_3_^+^ (SiO_2_NP–APTMS/TMAC), functionalized with primary amine (APTMS) and quaternary ammonium (TMAC) for DNA complexation and conjugation; (2) SiO_2_NP–N­(CH_3_)_3_^+^ /FITC (SiO_2_NP–TMAC/FITC), conjugated with FITC for fluorescence-based tracking; (3) SiO_2_NP–N­(CH_3_)_3_^+^ /PEG (SiO_2_NP–TMAC/PEG), functionalized with PEG to improve dispersion, enhance uptake, reduce cytotoxicity, shield DNA, prevent nonspecific binding, and facilitate DNA release from the complex; and (4) SiO_2_NP–N­(CH_3_)_3_^+^ /FITC/PEG (SiO_2_NP–TMAC/PEG/FITC), NPs labeled with both PEG and FITC, integrating the properties of positive charges, PEG, and FITC dye (Figure 10).

**Figure 10:**
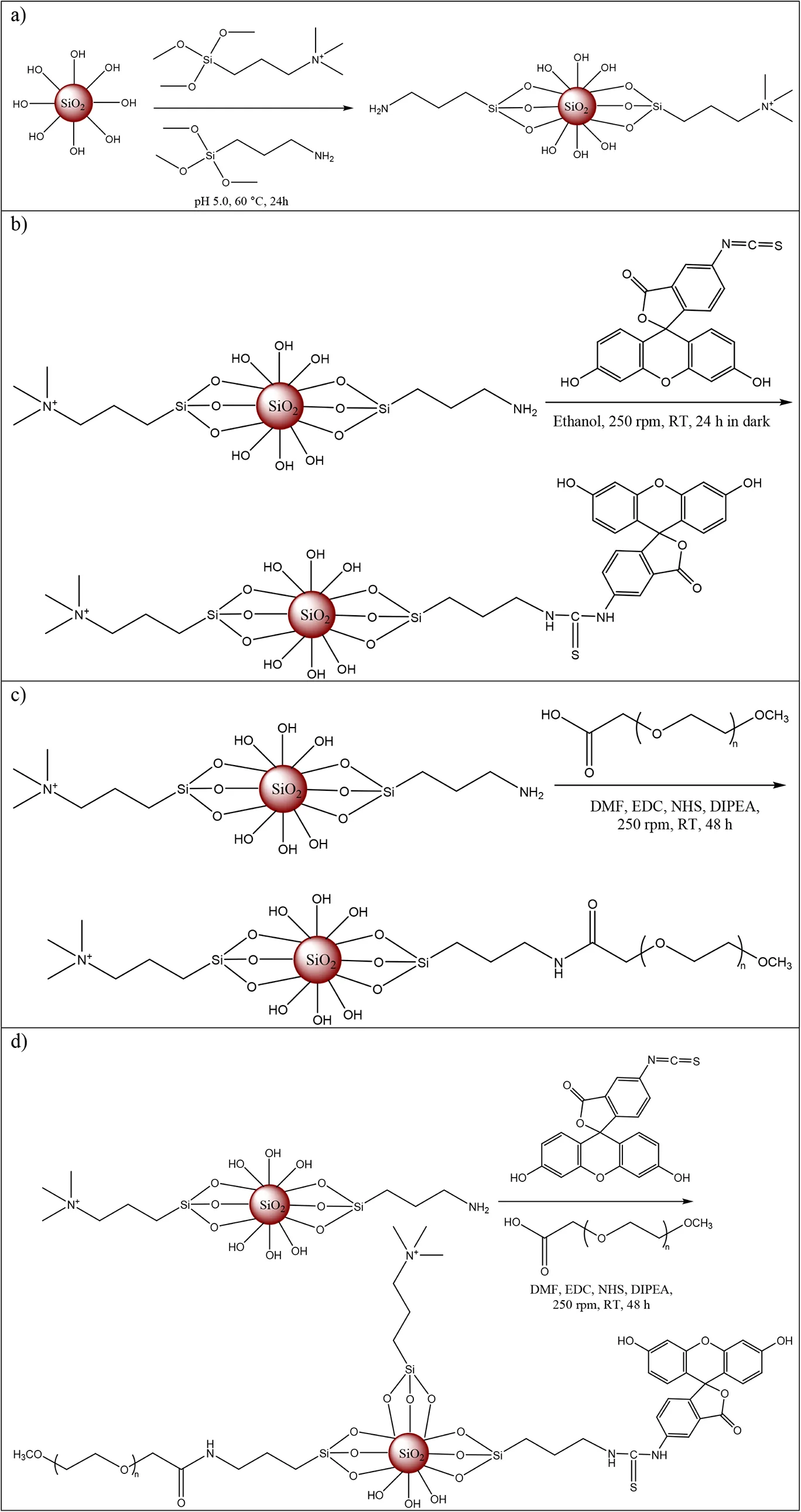
Schematic illustration of the stepwise surface functionalization of silica nanoparticles (SiO_2_NPs). a) Modification of SiO_2_NPs with APTMS and TMAC to generate positively charged SiO_2_NP-APTMS/TMAC nanoparticles. b) Conjugation of fluorescein isothiocyanate (FITC) to form SiO_2_NP–TMAC/FITC. c) PEGylation of SiO_2_NP-TMAC to produce SiO_2_NP–TMAC/PEG. d) Sequential functionalization with both PEG and FITC to generate multifunctional SiO_2_NP–TMAC/PEG/FITC nanoparticles.

#### 5.5.1 Synthesis and Characterization of Primary Amine (APTMS) and Quaternary Ammonium (TMAC) Terminal Mixed Dual Ligands Functionalized SiO_2_NP (SiO_2_NP-APTMS/TMAC)

The synthesis and characterization of SiO_2_NPs functionalized with dual ligands bearing primary amine and quaternary ammonium terminal groups were prepared as described in our recently published article.^39^ To begin the surface modification, 1 g of unmodified SiO_2_ NPs (Bare SiO_2_NPs (10–15 nm, Nissan Chemical, USA) was suspended in 9 mL of Milli-Q water and subjected to ultrasonic treatment at 25 °C for 15 min to ensure homogeneous dispersion. Without delay, two silane coupling agents—N-[3-(trimethoxysilyl)­propyl]-N,N,N-trimethylammonium chloride (2.133 g, 4.14 mmol) and (3-aminopropyl)­trimethoxysilane (0.361 mL, 2.07 mmol)—were simultaneously introduced into the dispersion.

The pH was carefully adjusted to 5 using 1 M hydrochloric acid, providing an optimal acidic environment for silanization. The reaction mixture was maintained under continuous stirring at 250 rpm and heated to 60 °C in an oil bath under reflux conditions for 24 h to facilitate covalent attachment of functional groups onto the silica surface.

Following the reaction, the aqueous phase was removed under reduced pressure using a rotary evaporator set to 60 °C. The resulting NP residue was redispersed in 15 mL of ethanol with a combination of stirring and brief sonication. To induce precipitation, 15 mL of *n*-hexane was then added to the ethanolic dispersion. The mixture was centrifuged at 4,400 rpm for 10 min to isolate the modified NPs from the solvent.

To ensure high purity, the isolated NPs underwent five sequential washing cycles. Each cycle involved redispersing the pellet in ethanol and precipitation with *n*-hexane, followed by centrifugation. An additional five washing cycles were carried out, this time using 10 mL of absolute ethanol for dispersion and 5 mL of *n*-hexane for precipitation, to thoroughly remove unreacted reagents and byproducts. The final NP pellet was redispersed in 15 mL of Milli-Q water and freeze-dried, yielding 1.2 g of a light, fluffy powder.

To investigate surface functionalization, the modified silica NPs were examined using attenuated total reflectance Fourier transform infrared spectroscopy (ATR-FTIR, Cary 600 series, Agilent), allowing identification of vibrational modes corresponding to chemical group transformations. Proton nuclear magnetic resonance (^1^H NMR) spectra were recorded in DMSO-*d*_6_ solvent using a Varian 600 MHz spectrometer to further confirm structural modifications at the molecular level. Elemental composition and chemical bonding states were analyzed via X-ray photoelectron spectroscopy (XPS) using a Kratos AXIS ULTRA system. Spectral data were acquired over a binding energy range of 0–1100 eV and subsequently interpreted using the CasaXPS software package. Thermal stability and organic content of the functionalized silica were assessed by thermogravimetric analysis (TGA, TA Instruments Q550). Measurements were conducted under a nitrogen atmosphere, with the temperature ramped from 25 to 800 °C at a constant rate of 10 °C per minute. Particle size distribution and zeta potential in aqueous media were determined by dynamic light scattering (DLS) using a Malvern Zetasizer Nano-ZS, enabling evaluation of colloidal behavior and surface charge. Finally, scanning electron microscopy (SEM, Hitachi S-5500) was employed to observe particle morphology and surface texture, providing complementary insights into the physical characteristics of the silica nanomaterials.^39^

#### 5.5.2 Synthesis and Characterization of Silica NPs Functionalized with Quaternary Ammonium Terminal Groups and Conjugated with FITC (SiO_2_NP–TMAC/FITC)

A total of 100 mg of SiO_2_NP–APTMS/TMAC was dispersed in 10 mL of absolute ethanol using ultrasonication for 10 min. In parallel, 39.8 mg (0.104 mmol) of fluorescein-5-isothiocyanate (5-FITC, AnaSpec, ≥ 95%) was dissolved in 10 mL of absolute ethanol under stirring in the dark. The FITC solution was then added to the NP dispersion, and the mixture was stirred in the dark for 24 h. Afterward, the reaction mixture was transferred to 50 mL centrifuge tubes and precipitated by adding 20 mL of *n*-hexane, followed by centrifugation at 4,400 rpm for 2 min. The resulting pellet was redispersed in 5 mL of ethanol, reprecipitated with 5 mL of hexane, and centrifuged at 4,400 rpm for 10 min. This washing cycle was repeated until the supernatant became colorless, indicating the removal of unbound FITC. The final pellet was redispersed in 10 mL of Milli-Q water and freeze-dried to yield 88 mg of fluffy orange powder. The conjugation of 5-FITC was confirmed by yellow color, green fluorescence, and ATR-FTIR spectroscopy. The hydrodynamic diameter and zeta potential were measured from a 1 mg/mL aqueous dispersion using Malvern Zetasizer Nano-ZS.

#### 5.5.3 Synthesis and Characterization of Silica NPs Functionalized with Quaternary Ammonium Terminal Groups and Conjugated with PEG (SiO_2_NP–TMAC/PEG)

A total of 100 mg of SiO_2_NP–APTMS/TMAC was dispersed in 5 mL of anhydrous DMF, followed by the sequential addition of 10.9 μL (0.078 mmol) N,N-diisopropylethylamine (DIPEA, ≥ 99%, Sigma-Aldrich), 130 mg of mPEG-Acetic Acid (1 kDa, Creative PEGWorks), 5.98 mg (0.052 mmol) *N*-hydroxysuccinimide (NHS, ≥ 97%, Sigma-Aldrich), and 9.97 mg (0.052 mmol) N-(3-(dimethylamino)­propyl)-N′-ethylcarbodiimide hydrochloride (EDC, commercial grade, Sigma-Aldrich). The mixture was stirred at room temperature for 48 h. After completion, the reaction mixture was transferred to 50 mL centrifuge tubes, precipitated with 30 mL of diethyl ether, and centrifuged at 4,400 rpm for 2 min. The resulting pellet was washed three times by resuspension in 5 mL of diethyl ether followed by centrifugation under the same conditions. The purified pellet was then redispersed in 5 mL of Milli-Q water, purged with nitrogen gas to remove residual ether, and transferred to a 10 kDa MWCO ultracentrifugal concentrator. The sample was centrifuged at 4,400 rpm for 20 min and the redispersion–centrifugation cycle was repeated ten times for complete purification. Finally, the particles were redispersed in 10 mL of Milli-Q water and lyophilized for 72 h, yielding 95 mg of white, fluffy powder. Successful conjugation of mPEG-Acetic Acid was confirmed by ATR-FTIR, and the hydrodynamic diameter along with the zeta potential were measured from a 1 mg/mL aqueous dispersion using a Malvern Zetasizer Nano-ZS.

#### 5.5.4 Synthesis and Characterization of Silica NPs with Quaternary Ammonium Terminal Groups and Conjugated with Both PEG and FITC (SiO_2_NP–TMAC/PEG/FITC)

The synthesis and workup of SiO_2_NP–TMAC/PEG/FITC followed the same procedure as that of SiO_2_NP–TMAC/PEG, with the exception that 9.95 mg (0.026 mmol) of 5-FITC was added to the reaction mixture along with the other reagents. Successful conjugation of mPEG–acetic acid and 5-FITC was confirmed by yellow color, green fluorescence, and ATR-FTIR. The hydrodynamic diameter and zeta potential were measured from a 1 mg/mL aqueous dispersion using a Malvern Zetasizer Nano-ZS.

### 5.6 The Cloning and Plasmid Isolation

The pBinGlyRed3 + 35S promoter plasmid (11,303 bp), which contains the DsRed fluorescent reporter gene under the control of the CaMV 35S promoter, was kindly provided by Dr. Edgar Cahoon (University of Nebraska–Lincoln) and was used for gene delivery in this study. The DsRed reporter enables direct visualization of transgene expression through red fluorescence. The plasmid was initially transformed into chemically competent *Escherichia coli* DH5α cells (New England BioLabs, Ipswich, MA) for amplification and long-term storage. The plasmid was extracted using the Qiagen Plasmid Maxi Kit (Qiagen, Hilden, Germany) according to the manufacturer’s instructions. For simplicity, this 11,303 bp construct is hereafter referred to as pDNA.

### 5.7 DNA-NP Complex Formation

Stock solutions of functionalized SiO_2_ NPs were prepared in deionized water at a concentration of 0.04 mg/mL. For PEGylated NPs (SiO_2_NP–TMAC/PEG and SiO_2_NP–TMAC/PEG/FITC), the suspension was sonicated for 45 min at room temperature using a standard ultrasonic bath to ensure complete dispersion. The NP solution was then mixed with pDNA (0.5 μg/μL) prepared in nuclease-free water. A total volume of 200 μL of the DNA–NP mixture was incubated at room temperature for 2 h to allow for complete complex formation prior to transformation.

### 5.8 Gel Retardation Assay

To determine the optimal ratio for pDNA binding to functionalized SiO_2_NPs, a gel retardation assay was performed. The pDNA was mixed with functionalized SiO_2_NPs at varying DNA:NP weight ratios of 1:1, 1:2, 1:3, 1:4, 1:5, and 1:8. Following incubation, samples were loaded onto a 1% agarose gel prepared with Tris-acetate-EDTA (TAE) buffer and run at 100 V for ∼45 min. Free (unbound) DNA migrated through the gel, whereas DNA bound to NPs remained in the loading wells due to impaired mobility. Gels were stained with ethidium bromide (or SYBR Safe) and visualized under UV illumination to assess complexation efficiency. The ratio showing complete retardation (i.e., no visible migration of free DNA) was selected as the optimal binding condition for subsequent transformation experiments.

### 5.9 Transformation of pDNA-Functionalized SiO_2_NP Complex Solution into Canola Protoplasts and Microspores

Protoplast suspensions were adjusted to approximately 1 × 10^6^ cells/mL in 0.5 M mannitol. DNA-functionalized SiO_2_NP (DNA–NP) complexes, prepared at optimized DNA:NP binding ratios, were added directly to the protoplast suspensions under gentle shaking at room temperature (Figure 11). To evaluate uptake efficiency, protoplasts were incubated with DNA–NP complex for 1, 4, and 24 h post-transformation.

**Figure 11:**
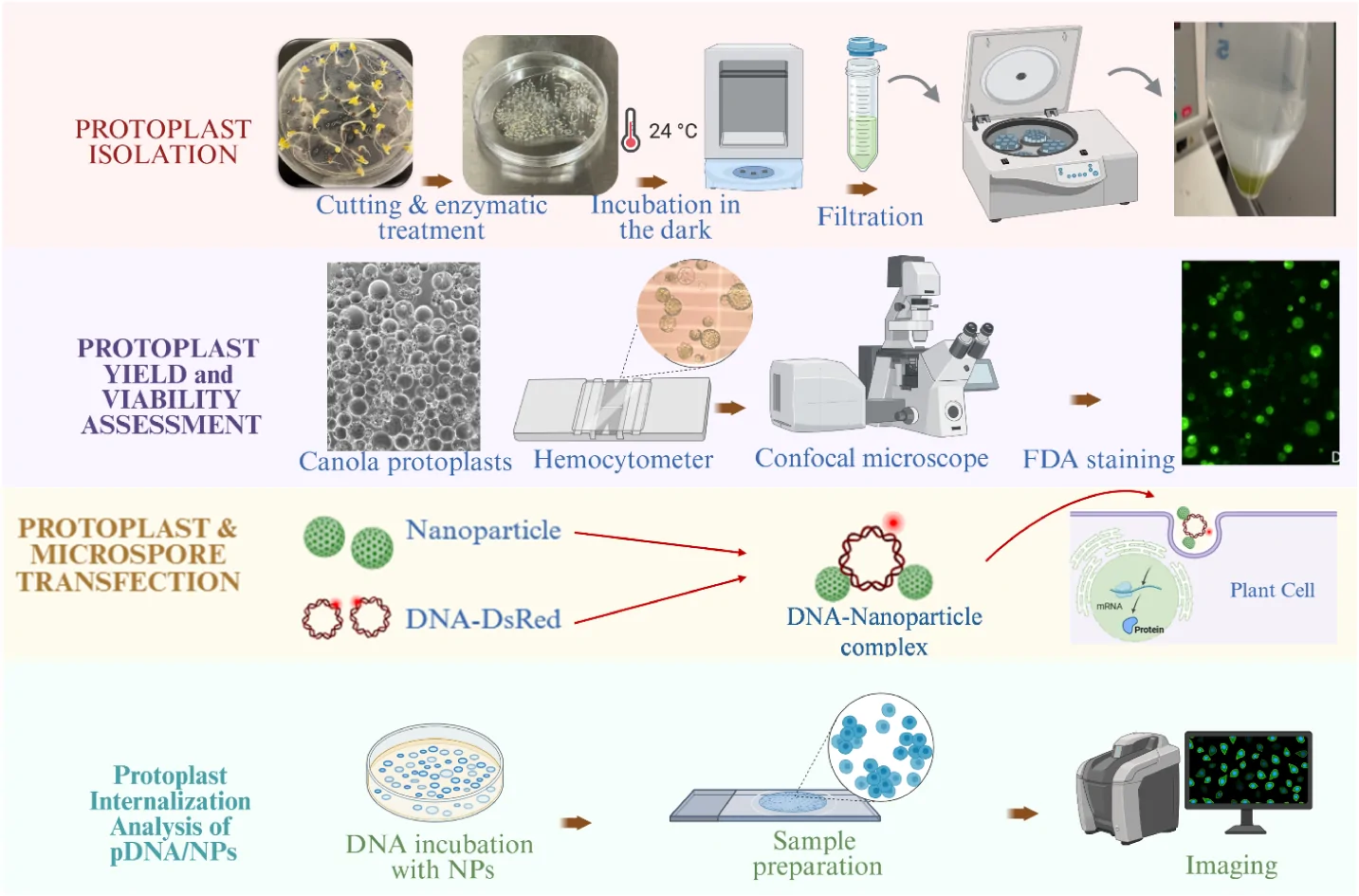
Schematic overview of the transient gene delivery workflow in canola protoplasts. The workflow includes protoplast isolation from hypocotyl and leaf tissues, assessment of protoplast yield and viability, formation of pDNA-functionalized SiO_2_ nanoparticle complexes, protoplast transfection, and evaluation of nanoparticle uptake and DsRed expression by fluorescence imaging. Created with BioRender.com.

Freshly isolated microspore suspensions were adjusted to 4 × 10^4^ cells/mL in NLN-13 medium. DNA–NP complexes prepared at optimized binding ratios were added to the microspore suspensions and incubated under gentle shaking at room temperature (Figure 12). Due to the higher sensitivity of microspores compared to protoplasts, shorter exposure times of 30 and 60 min were selected to promote NP uptake while minimizing cellular stress.

**Figure 12:**
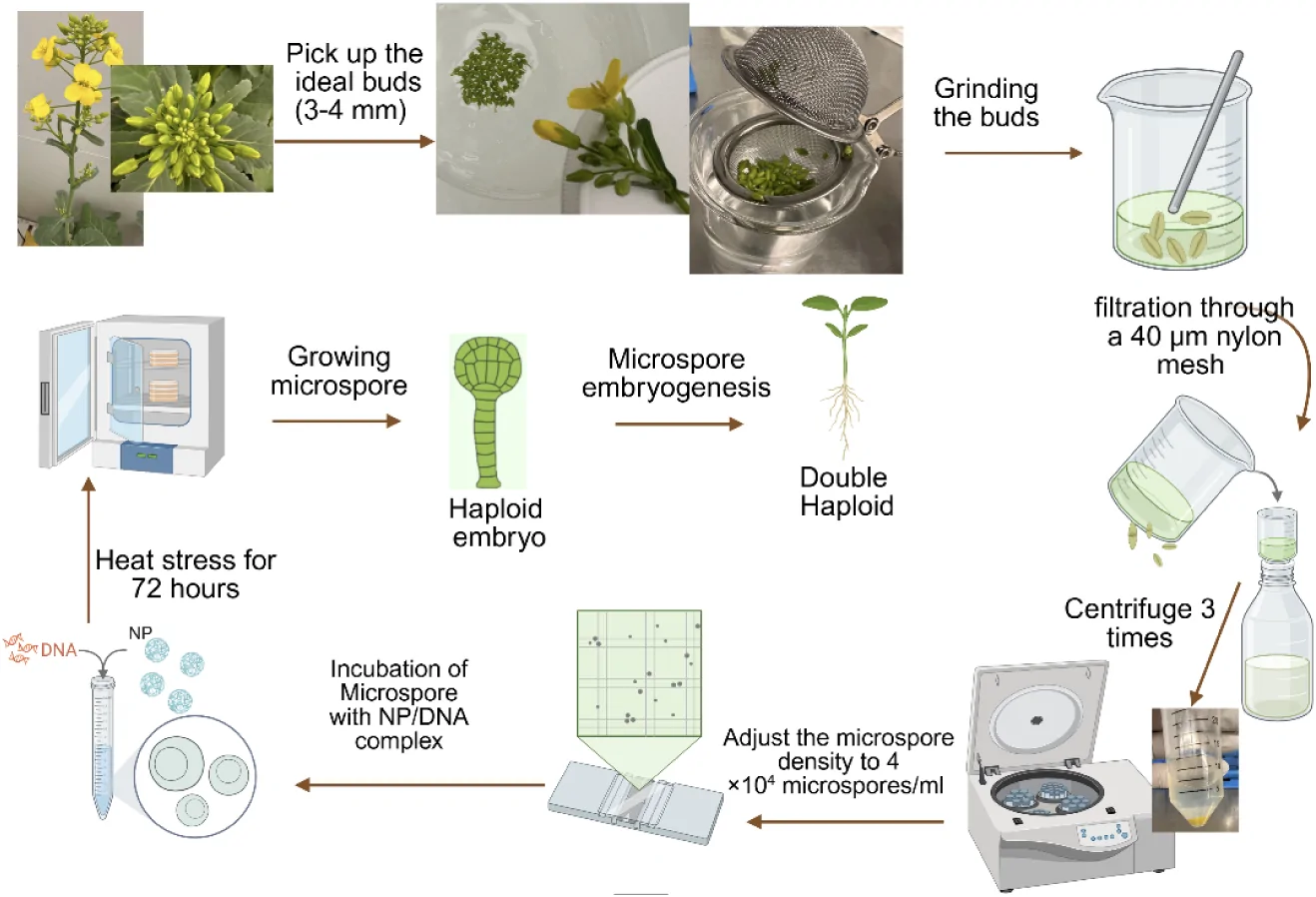
Schematic overview of nanoparticle-mediated transformation and regeneration of canola microspores. The workflow illustrates selection of suitable flower buds (3–4 mm), isolation and purification of microspores, incubation with pDNA-functionalized SiO_2_ nanoparticle complexes, induction of microspore embryogenesis by heat treatment, embryo development, and regeneration of doubled haploid plants. Created with BioRender.com.

### 5.10 Fluorescence Observation and Imaging

After incubation with DNA–NP complexes, protoplasts and microspores were gently washed with fresh culture medium to remove excess NPs and unbound DNA. Fluorescence imaging was performed 48 h after transformation using an Olympus FLUOVIEW FV3000 confocal laser scanning microscope equipped with a FITC filter to visualize the penetration and localization of the DNA–NP complexes. FITC-labeled NPs were detected using an excitation wavelength of 488 nm and emission collection between 500 and 550 nm (green channel), while DsRed fluorescence from the expressed reporter gene was observed using an excitation wavelength of 561 nm and emission detection between 570 and 620 nm (red channel). Images were captured using both brightfield and fluorescence channels to assess cell morphology, NP internalization, and transgene expression. All imaging parameters were kept constant across samples to allow for qualitative comparison.

### 5.11 Molecular Confirmation of Regenerated Canola Plants by PCR

To verify the presence of the delivered transgene, genomic DNA was extracted from young leaves of four regenerated canola plantlets obtained following nanoparticle-mediated gene delivery. Approximately 0.2 g of fresh leaf tissue from each regenerated plant was collected, and genomic DNA was isolated using the DNeasy Plant Maxi Kit (Qiagen GmbH, Hilden, Germany) according to the manufacturer’s instructions.

PCR amplification of the DsRed reporter gene was performed using the primer pair DsRed-fw (5′-CCTCCTCCGAGAACGTCATC-3′) and DsRed-rv (5′-TTGGTACCAGGAACAGGTGG-3′). PCR reactions were carried out using Promega PCR Master Mix (Promega, Madison, WI, USA). The amplification program consisted of an initial denaturation step at 94 °C for 5 min, followed by 35 cycles of denaturation at 94 °C for 1 min, annealing at 60 °C for 1 min, and extension at 72 °C for 1 min, with a final extension at 72 °C for 5 min.

PCR products were separated on a 1% agarose gel (SYBR Safe DNA gel stain, Thermo Fisher) and visualized under UV illumination. The expected amplicon size for the DsRed fragment was confirmed by comparison with a DNA ladder and a positive plasmid control.

### 5.12 Evaluation of Transformation Efficiency

Transformation efficiency was assessed by monitoring DsRed fluorescence signal in protoplasts and microspores following incubation with DNA–NP complexes. Cells were imaged using the confocal microscope at multiple time points post-transformation (typically 2 to 7 days). Red fluorescence, indicative of DsRed gene expression, was used as a marker for successful gene delivery and expression. The percentage of transformed cells was calculated by counting the number of red-fluorescent cells relative to the total number of viable cells in each field of view:

Transformation efficiency(%)=Total number of DsRed‐positive cells/Total number of viable
cells×100

Each sample was evaluated by at least three biological replicates, and 5–10 random fields were imaged per replicate to ensure representative quantification. Controls included untreated protoplasts or microspores and the same samples incubated with NPs alone (without DNA) to rule out autofluorescence and NP-related artifacts.

### 5.13 Statistical Analysis

To evaluate cell viability, quantitative viability data were collected from three biological replicates. Statistical analyses were performed using GraphPad Prism version 9.5. Results are presented as mean ± standard deviation (SD). Statistical significance was determined using one-way ANOVA followed by appropriate post hoc multiple-comparison testing. Differences were considered statistically significant at *p* < 0.05. Each experiment was independently repeated three times.

## Data Availability

All data generated or analyzed during this study are included in this published article.

## Abbreviations

NPs: nanoparticles
SiO_2_NPs: silica nanoparticles
SSiO_2_NPs: solid silica nanoparticles
PEG: polyethylene glycol
FITC: fluorescein isothiocyanate
FDA: fluorescein diacetate
pDNA: plasmid DNA
